# Long-Term Nitrogen Addition and Reduced Precipitation Alter Seasonal Soil N_2_O Fluxes and N-Transformation Processes in a Temperate Forest

**DOI:** 10.3390/plants15213436

**Published:** 2026-11-04

**Authors:** Peng Qu, Yajuan Xing, Guancheng Liu, Lijian Xu, Qinggui Wang

**Affiliations:** 1 School of Life Sciences, Heilongjiang University, Harbin 150080, China; njzyqupeng2008@163.com (P.Q.); xulijian@hlju.edu.cn (L.X.); 2 School of Life Sciences, Qufu Normal University, Qufu 273165, China; lxtxlgc123@126.com

**Keywords:** soil N_2_O flux, nitrogen addition, reduced precipitation, dissolved organic carbon stoichiometry, N-transformation enzyme indicators, temperate forest

## Abstract

Nitrous oxide (N_2_O) is a potent greenhouse gas, and forest-soil N_2_O emissions are highly sensitive to long-term changes in nitrogen deposition and precipitation regimes. However, how long-term nitrogen addition interacts with reduced precipitation to regulate seasonal soil N_2_O flux and its associated environmental and process-related factors remains poorly understood in temperate forests. We conducted a 14-year field experiment in a temperate broadleaved–Korean pine forest with four treatments: control (CK), nitrogen addition (NA; 50 kg N ha^−1^ yr^−1^), precipitation reduction (PR; 30% throughfall exclusion), and their combination (NAPR). Soil N_2_O flux was monitored continuously throughout May, July, and September 2023; daily mean fluxes were calculated from the continuous measurements and then averaged to obtain monthly means. Soil properties, inorganic N, dissolved organic carbon (DOC) stoichiometry, and N-transformation-related enzyme indicators were measured once per monitoring month. Reduced precipitation markedly decreased monthly mean N_2_O flux, with the largest treatment divergence occurring in July. Across all three monitoring periods, N_2_O flux consistently followed the order NA > CK > PR > NAPR. Under ambient precipitation, N addition increased N_2_O flux, whereas under reduced precipitation it further decreased N_2_O flux relative to PR. Nitrogen addition substantially increased NH_4_^+^–N, NO_3_^−^–N, and total inorganic N under both precipitation regimes, but the particularly low N_2_O flux under NAPR persisted. Redundancy analysis and correlation analyses showed coordinated variation among soil environmental and resource conditions and N-transformation-related indicators, while random forest analysis identified urease (UE) and ammonia monooxygenase (AMO) among the highest-ranked predictors and showed robust predictive performance under grouped cross-validation. Overall, reduced precipitation reversed the stimulatory effect of long-term nitrogen addition on N_2_O flux, with the treatment response associated with soil moisture, substrate availability, and N-transformation-related indicators rather than attributable to a single measured pathway.

## 1. Introduction

Soil nitrous oxide (N_2_O) is a potent greenhouse gas and an ozone-depleting substance. Its 100-year global warming potential is approximately 273 times that of CO_2_. Changes in N_2_O emissions therefore have important implications for climate feedbacks from terrestrial ecosystems [1]. Forest soils are an important terrestrial source of N_2_O and are highly sensitive to changes in nitrogen input and hydroclimatic conditions [2,3]. In forest ecosystems, global change is simultaneously altering water and nutrient regimes. Increasing drought frequency, greater hydrological variability, and shifts in precipitation patterns are changing soil moisture conditions. Meanwhile, anthropogenic activities continue to increase atmospheric inputs of reactive nitrogen, thereby modifying ecosystem nitrogen cycling and nutrient balance [1,4,5]. Understanding how net soil N_2_O flux responds to the combined changes in water availability and nitrogen input is therefore essential for predicting climate feedbacks from forest soils.

Net soil N_2_O flux should not be viewed simply as a direct emission response to increased substrate availability. Instead, it reflects the combined outcome of N_2_O-producing and N_2_O-reducing processes. N_2_O can be produced during nitrification and denitrification, including their coupled pathways. By contrast, terminal N_2_O reduction within denitrification converts N_2_O to N_2_ and thereby lowers net emissions [2,6]. Net N_2_O flux therefore depends not only on substrate availability, but also on how the relative contributions of N_2_O production and terminal reduction shift under changing environmental conditions [7]. This balance is especially important in temperate forests. During the growing season, marked variation in temperature, precipitation, and soil moisture can alter oxygen diffusion, redox heterogeneity, and rainfall-pulse-driven microsite conditions.

Within this framework, reduced precipitation is expected to shift the balance between N_2_O production and reduction by constraining soil moisture and altering oxygen diffusion, redox heterogeneity, and substrate transport. As a result, it often lowers net N_2_O emissions or weakens emission peaks [8,9]. However, this response is not uniform. Its magnitude and direction can vary with climatic background, soil properties, and drought intensity [10,11]. By contrast, nitrogen addition usually increases the availability of NH_4_^+^ and NO_3_^−^ and may therefore enhance the potential for N_2_O production. However, this stimulation is not universal. It can be constrained by soil moisture, redox conditions, carbon supply, and the efficiency of terminal N_2_O reduction [12,13]. Under long-term nitrogen enrichment combined with reduced precipitation, the response of net N_2_O flux may not be simply additive. For temperate forests, one key unresolved question is whether increased inorganic nitrogen availability under reduced precipitation leads to higher net soil N_2_O flux, and why this response is often constrained.

Addressing this question requires consideration not only of water availability and inorganic nitrogen supply, but also of the broader soil physicochemical background and dissolved C–N stoichiometry. Soil water content, pH, soil organic carbon, and available phosphorus shape the microenvironment in which microbial nitrogen transformations occur. pH may also influence the efficiency of terminal N_2_O reduction and the overall expression of N_2_O emissions [14,15]. In parallel, DOC-based stoichiometric indices such as DOC:DON, DOC:NH_4_^+^, and DOC:NO_3_^−^ reflect the matching between dissolved carbon and nitrogen resources [16,17,18]. They may therefore influence how N_2_O production and reduction are coupled. These properties help define the environmental and stoichiometric context in which N_2_O-producing and N_2_O-reducing processes are linked. However, under long-term nitrogen enrichment combined with reduced precipitation, it remains unclear whether these physicochemical and stoichiometric properties directly regulate net N_2_O flux or primarily shape the environmental context in which subsequent N-transformation processes occur [19,20,21,22,23,24].

To explore the process-related basis of net soil N_2_O responses to long-term nitrogen enrichment and reduced precipitation, N-transformation enzyme-related indicators provide a useful entry point. Ammonia monooxygenase (AMO) is closely associated with the conversion of NH_4_^+^ to NO_2_^−^ and can therefore provide an indicator of nitrification-related processes potentially contributing to N_2_O production [25]. By contrast, nitrous oxide reductase (N_2_OR) mediates the terminal reduction of N_2_O to N_2_ and therefore provides an indicator related to the sink side of the soil N_2_O budget [26]. From this perspective, a key question is whether seasonal variation in net soil N_2_O flux is associated with contrasting responses of AMO- and N_2_OR-related processes under changing soil moisture and substrate conditions. Under reduced precipitation, long-term nitrogen addition may alter inorganic nitrogen availability and dissolved C–N stoichiometry while soil moisture remains constrained, thereby reshaping enzyme-related process capacities across seasons [10,27]. Although previous studies have separately documented the effects of nitrogen addition and altered precipitation on soil N_2_O emissions, a coherent explanation for their combined long-term effects in temperate forests is still lacking. In a previous 2-year field study conducted in a temperate broad-leaved Korean pine forest, precipitation reduction alone decreased soil N_2_O emissions, whereas the combined treatment stimulated emissions relative to the control and nitrogen addition alone [28]. However, it remains unclear whether a similar response is observed after long-term experimental manipulation, and whether increased inorganic nitrogen availability under reduced precipitation is consistently accompanied by higher net N_2_O flux. It is also unclear how DOC stoichiometry contributes to this response and how variation in AMO- and N_2_OR-related indicators is associated with net N_2_O flux under contrasting water and nitrogen conditions. Together, these uncertainties point to the need for an integrated analysis linking environmental conditions, substrate availability, N-transformation indicators, and net N_2_O flux under combined nitrogen addition and reduced precipitation. To address these questions, we used a 14-year field manipulation experiment in a temperate forest with four treatments: control (CK), nitrogen addition (NA), precipitation reduction (PR), and nitrogen addition combined with precipitation reduction (NAPR). During three monitoring periods in May, July, and September, representing spring, summer, and autumn, respectively, we quantified soil N_2_O fluxes, soil physicochemical properties, inorganic nitrogen availability, DOC stoichiometry, and enzyme-related indicators associated with nitrogen transformation. We then integrated univariate and multivariate analyses to characterize treatment- and season-dependent responses and their associations with N_2_O variation. We tested three hypotheses: (1) reduced precipitation decreases net soil N_2_O flux across the three seasonal monitoring periods, and long-term N addition under reduced precipitation is insufficient to offset this decline; (2) inorganic N availability and DOC stoichiometry are associated with N_2_O variation within a broader environmental and resource context rather than acting as dominant standalone controls; and (3) N-transformation-related indicators exhibit season-dependent responses to N addition and reduced precipitation, with their relationships to net N_2_O flux varying across the treatment–season environmental gradient.

## 2. Materials and Methods

### 2.1. Study Site

The study site is located in a mixed broadleaved–Korean pine (*Pinus koraiensis*) forest within the Changbai Mountain National Nature Reserve, Jilin Province, northeastern China (42°24′18″ N, 128°6′19″ E; 750–770 m a.s.l.). The forest is an old-growth mixed stand dominated by *Pinus koraiensis*, *Tilia amurensis*, and *Fraxinus mandshurica*, with a multilayered structure and a stand age exceeding 300 years. The soil is classified as Eutric Cambisol (FAO), developed from volcanic ash parent material and characterized by high organic matter content and good drainage. The 0–10 cm soil layer in the control plots had mean total carbon (TC) and total nitrogen (TN) contents of 61.22 and 3.94 g kg^−1^, respectively, and a TC:TN ratio of 15.77 (Table 1). The region has a temperate continental monsoon climate, with a mean annual air temperature (MAT) of 3.6 °C and a mean growing-season air temperature of 15 °C. Mean annual precipitation (MAP) is 855 mm, with more than 80% occurring between May and August. Long-term meteorological records indicate a declining trend in MAP in the Changbai Mountain region, accompanied by an increasing tendency toward drought conditions in northeastern China, particularly in autumn, indicating elevated regional drought risk [29,30,31].

### 2.2. Experimental Design and Soil Sampling

**Figure 1:**
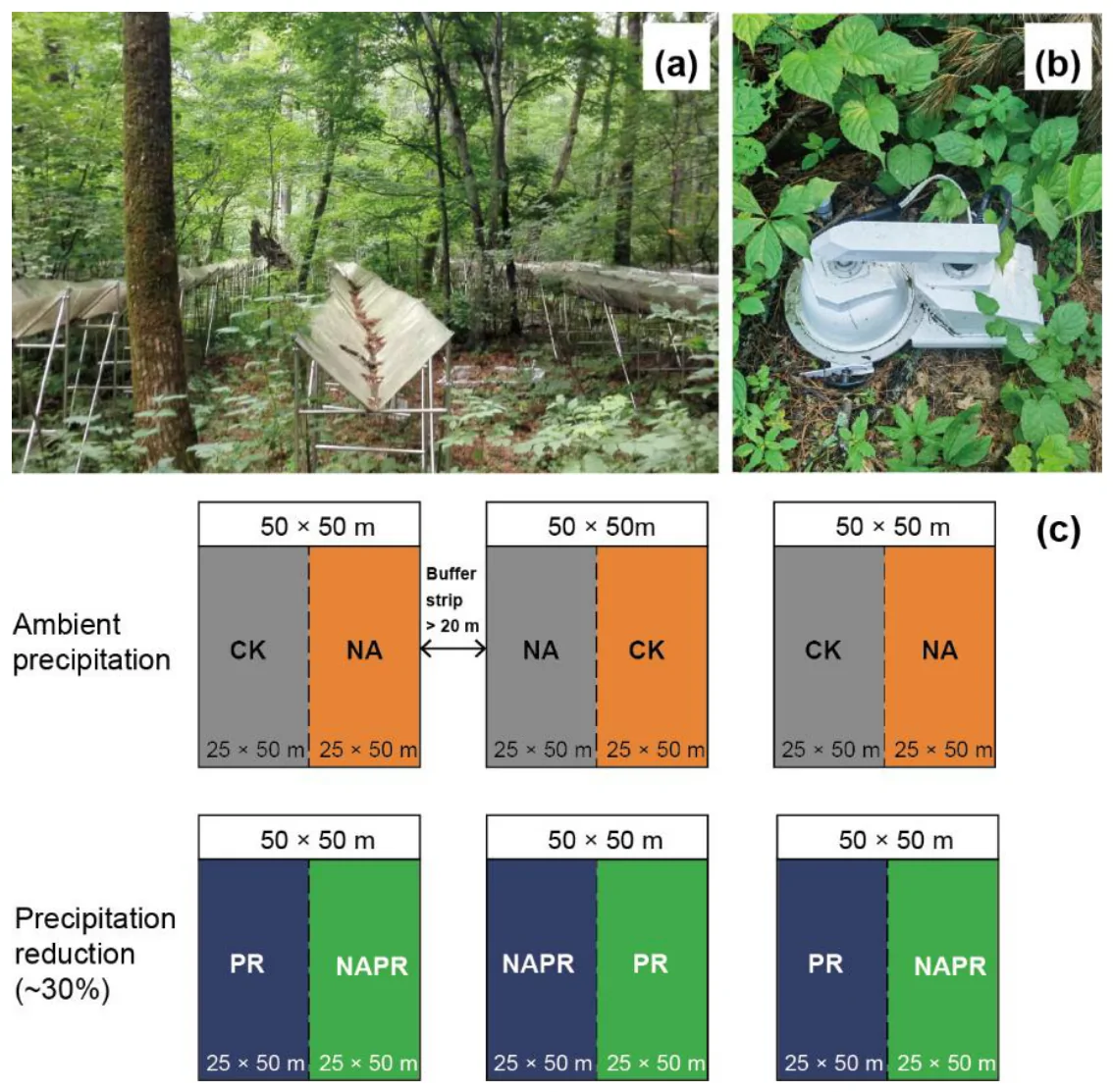
Experimental setup and N_2_O flux monitoring system: (**a**) throughfall-reduction panels, (**b**) automated N_2_O monitoring system, and (**c**) split-plot design.

In 2009, a long-term nitrogen addition and precipitation reduction experiment was established using a split-plot design. Six 50 × 50 m main plots were randomly established, with buffer strips of >20 m between adjacent plots (Figure 1a,c). Three main plots were assigned to ambient precipitation and three to precipitation reduction. Each main plot was divided into two 25 × 50 m subplots by inserting a barrier sheet approximately 50 cm into the soil to minimize lateral nutrient movement between adjacent subplots. Within each main plot, the N-addition and no-N subplots were randomly assigned. Thus, precipitation reduction was applied at the main-plot level and nitrogen addition at the subplot level. Four treatment combinations were established, with three replicates per treatment: natural precipitation control (CK), nitrogen addition (NA), precipitation reduction (PR), and nitrogen addition combined with precipitation reduction (NAPR).

To implement PR, V-shaped transparent polycarbonate panels (approximately 95% light transmittance) were installed approximately 1 m above the forest floor to intercept about 30% of ambient throughfall (approximately 210 mm yr^−1^) while maintaining adequate air circulation [32,33]. At the end of the growing season, the panels were inverted to prevent snow-load damage and allow natural snowfall. Nitrogen was applied as an NH_4_NO_3_ solution at a rate of 50 kg N ha^−1^ yr^−1^, approximately twice the historical atmospheric N deposition in the region [34]. Applications were performed monthly from May to October each year. For each application, NH_4_NO_3_ was dissolved in 40 L of deionized water, and the CK and PR subplots received the same volume of deionized water without NH_4_NO_3_ to control for water input associated with fertilization.

Soil sampling was conducted once each in May, July, and September 2023, representing the spring, summer, and autumn monitoring periods, respectively. At each sampling event, the surface litter layer was carefully removed, and the soil corer was pre-conditioned with site soil to minimize cross-sample contamination. Ten soil cores (0–10 cm depth; 5 cm inner diameter) were randomly collected from each subplot and combined into a single composite sample. Samples were immediately transported to the laboratory under cooled conditions and analyzed upon arrival.

### 2.3. Laboratory Analysis of Soil Properties

Soil water content (SWC) was determined gravimetrically by oven-drying fresh soil at 105 °C for 24 h and expressed on a dry-mass basis (%). Soil pH was measured using a pH meter in a soil–deionized water suspension (*w*/*v* = 1:2.5; PHS-3E, Shanghai INESA, Shanghai, China). Total carbon (TC) was determined by dry combustion, whereas total nitrogen (TN) and total phosphorus (TP) were measured following H_2_SO_4_–H_2_O_2_ digestion. Soil organic carbon (SOC) was determined after pretreatment with 0.1 mol L^−1^ HCl to remove inorganic carbon. For fresh soil samples, inorganic N (NH_4_^+^-N, NO_3_^−^-N, and NO_2_^−^-N) was extracted with 2 mol L^−1^ KCl (*w*/*v* = 1:10). Dissolved organic carbon (DOC) and dissolved organic nitrogen (DON) were extracted with 0.5 mol L^−1^ K_2_SO_4_ (*w*/*v* = 1:5), and available phosphorus (AP) was extracted with 0.5 mol L^−1^ NaHCO_3_ (*w*/*v* = 1:5). Concentrations of TC, SOC, DOC, and DON were quantified using a TC/TN analyzer (multi N/C 3100, Analytik Jena AG, Jena, Germany). Concentrations of NH_4_^+^-N, NO_3_^−^-N, NO_2_^−^-N, TN, TP, and AP were quantified using a continuous-flow analyzer (AA3, Bran-Luebbe, Hamburg, Germany). Total inorganic nitrogen (TIN) was calculated as the sum of NH_4_^+^-N, NO_3_^−^-N, and NO_2_^−^-N.

### 2.4. Measurement of Soil N_2_O Flux and Environmental Variables

Soil N_2_O flux was continuously monitored throughout May, July, and September 2023, representing the spring, summer, and autumn monitoring periods, respectively (Figure 1b). Three independent monitoring channels were used for each treatment, with one channel located in each of the three replicate subplots. N_2_O fluxes were measured using an SF-3500 automatic multi-channel soil gas flux measurement system (Beijing LICA United Technology Co., Ltd., Beijing, China) equipped with an LGR-ICOS GLA151-N2OM1 N_2_O/CH_4_ analyzer (ABB, Quebec City, QC, Canada). According to the manufacturer’s specifications, the N_2_O measurement precision (1σ) was 0.5 ppb at a 1-s averaging time and 0.2 ppb at a 10-s averaging time for concentrations below 500 ppb. The system continuously recorded N_2_O concentrations, soil temperature (ST), and air temperature. Fluxes were calculated from the linear rate of change in chamber N_2_O concentration over time. The SF-3500 software automatically accepted flux estimates only when the linear fit had an R^2^ > 0.90; fits below this threshold were not output as valid flux records. Valid flux records were averaged to obtain daily mean N_2_O fluxes, from which monthly mean fluxes were calculated for May, July, and September. Monthly mean N_2_O fluxes were used in analyses relating N_2_O to soil physicochemical properties and enzyme-related indicators. Precipitation data for 2023 were obtained from the ERA5-Land reanalysis dataset [35].

### 2.5. Nitrogen Transformation-Related Enzyme Indicators

Indicators of seven N-transforming enzymes were quantified, including ammonia monooxygenase (AMO), nitrite oxidoreductase (NXR), nitrate reductase (NR), nitrite reductase (NIR), nitric oxide reductase (NOR), nitrous oxide reductase (N_2_OR), and urease (UE). Potential activities of NR, NIR, and UE were determined using microplate-based colorimetric assay kits (Keming Biotechnology, Suzhou, China). AMO, NXR, NOR, and N_2_OR were quantified using commercial enzyme-linked immunosorbent assay (ELISA) kits (Jiangsu Meimian Industrial Co., Ltd., Yancheng, China) according to the manufacturer’s instructions and were expressed as U l^−1^, where U denotes the enzyme activity unit specified by the manufacturer. All seven measurements were treated as N-transformation enzyme-related indicators associated with nitrification, denitrification, and N mineralization.

### 2.6. Data Analysis

We used linear mixed-effects models (LMMs) to test the responses of monthly mean N_2_O flux, soil physicochemical properties, and N-transformation enzyme-related indicators to nitrogen addition (NA), reduced precipitation (PR), season, and their interactions. The hierarchical model accounted for the split-plot and repeated-measures design by including main plot and subplot as random intercepts, with repeated seasonal observations occurring within subplots. N_2_O was natural-log-transformed prior to analysis, whereas all other variables were analyzed on their original scales. Fixed effects were evaluated using Type III ANOVA, and post hoc comparisons were performed using estimated marginal means with Tukey adjustment.

Redundancy analysis (RDA) was used to examine relationships between soil environmental variables and enzyme-related indicators. The enzyme-related indicator matrix was used as the response matrix, and SWC, ST, pH, SOC, AP, DOC:DON, and DOC:NO_3_^−^ were included as explanatory variables. All variables were z-score standardized prior to analysis, and monthly mean N_2_O flux was fitted as a supplementary vector. Model significance was assessed by permutation tests, with plot used as a blocking factor. Spearman correlation analyses were additionally used to characterize pairwise associations among soil environmental/resource variables and between these variables and N-transformation-related enzyme indicators. To assess whether pooled enzyme–N_2_O associations persisted after accounting for season and repeated observations, separate linear mixed-effects models were fitted for each enzyme indicator, with season as a fixed effect and plot as a random intercept (Table S8). Random forest (RF) analysis was used to evaluate the predictive importance of variables associated with monthly mean N_2_O flux. The model included nine predictors—SWC, pH, SOC, DOC:DON, DOC:NO_3_^−^, UE, AMO, NIR, and N_2_OR—with untransformed monthly mean N_2_O flux as the response. The model was fitted with 2000 trees, mtry = 3, and nodesize = 5. Variable importance was expressed as the percentage increase in mean squared error (%IncMSE) and evaluated using permutation analysis. Predictive performance was assessed using grouped cross-validation. In the primary leave-one-main-plot-out validation, the paired N-addition and no-N subplots within each precipitation main plot, including all seasonal observations, were withheld together to prevent observations from the same experimental unit from occurring in both training and validation sets. Model performance was evaluated using R^2^, RMSE, and MAE. All analyses were conducted in R 4.2.2.

## 3. Results

### 3.1. Seasonal Dynamics and Treatment Effects on Soil N_2_O Fluxes

**Figure 2:**
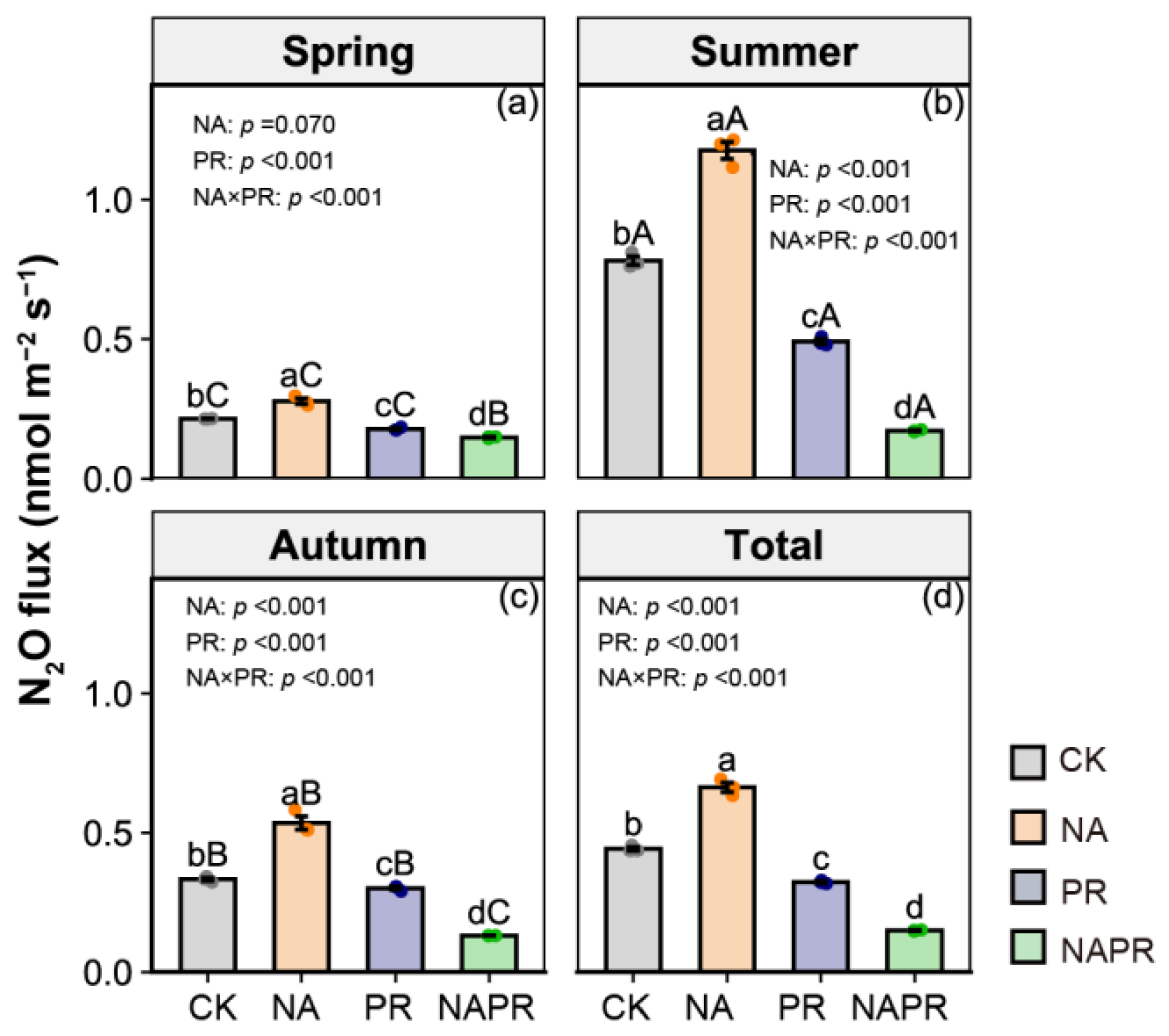
Effects of long-term nitrogen addition and reduced precipitation on soil N_2_O flux across sampling seasons. (**a**) Spring; (**b**) Summer; (**c**) Autumn; (**d**) Total. Bars represent mean soil N_2_O flux ± standard error (SE; *n* = 3), and points show individual subplot-level observations. Different lowercase letters indicate significant differences among treatments within the same sampling season, whereas different uppercase letters indicate significant differences among sampling seasons within the same treatment (Tukey-adjusted multiple comparisons, *p* < 0.05). *P* values in the upper-left corner of each panel indicate the effects of nitrogen addition (NA), precipitation reduction (PR), and their interaction (NA × PR). For Total, bars and points represent the mean N_2_O flux across spring, summer, and autumn within each subplot and should not be interpreted as cumulative growing-season N_2_O emission.

Design-based split-plot tests showed significant effects of N addition, precipitation reduction, season, and all interaction terms on soil N_2_O flux, including significant NA × PR and NA × PR × Season interactions (all *p* < 0.001; Table S2). Across all three monitoring periods, model-estimated N_2_O flux consistently followed the order NA > CK > PR > NAPR (Figure 2; Table 2). Seasonal variation was pronounced, with the highest fluxes occurring in summer, while daily records further showed marked within-month temporal variation in N_2_O flux alongside changing precipitation and air temperature conditions (Figure S1). Under ambient precipitation, N addition increased N_2_O flux relative to CK by 29.6% in spring, 50.5% in summer, and 60.3% in autumn (Table S1). In contrast, under reduced precipitation, N addition further decreased N_2_O flux relative to PR by 17.1%, 65.2%, and 56.5% in spring, summer, and autumn, respectively. Precipitation reduction alone decreased N_2_O flux relative to CK by 17.0% in spring, 37.2% in summer, and 9.6% in autumn. Consequently, N_2_O flux under NAPR was 31.2%, 78.1%, and 60.6% lower than under CK in spring, summer, and autumn, respectively. Treatment divergence was greatest in summer, when the back-transformed estimated marginal means were 1.176, 0.781, 0.491, and 0.171 nmol m^−2^ s^−1^ for NA, CK, PR, and NAPR, respectively (Table 2). These results demonstrate that the stimulatory response to N addition observed under ambient precipitation was reversed under reduced precipitation, and that additional N input did not offset the precipitation-reduction-induced suppression of soil N_2_O flux.

### 3.2. Seasonal and Treatment Effects on Soil Physicochemical Properties

Soil physicochemical properties responded differentially to season, treatment components, and their interactions, although response patterns varied among variables (Table 3; Tables S2 and S3). Among these variables, SWC showed a strong and consistent response to reduced precipitation, remaining significantly lower under PR and NAPR than under CK across seasons (Table 3). Relative to CK, mean SWC under PR was lower by 8.1, 6.5, and 9.0 percentage points in spring, summer, and autumn, respectively, whereas the corresponding reductions under NAPR were 10.9, 9.0, and 14.2 percentage points. ST (soil temperature) exhibited a clear seasonal pattern, peaking in summer and declining in spring and autumn. Soil pH also responded clearly to N addition and season, with generally higher values under CK and PR than under NA and NAPR, particularly in summer and autumn (Table 3). SOC showed a treatment-dependent seasonal response and was generally highest under NA (Table 3). AP also varied strongly with season and reached its highest values in summer. Treatment differences were most pronounced in summer, with NA showing the highest values and NAPR the lowest (Table 3). In addition, TC, TN, TP, and their stoichiometric ratios (TC:TN, TC:TP, and TN:TP) also varied with season and treatment components, although their significance patterns were not fully consistent among variables (Table S3).

### 3.3. Responses of Soil Inorganic N Availability and DOC Stoichiometry

**Figure 3:**
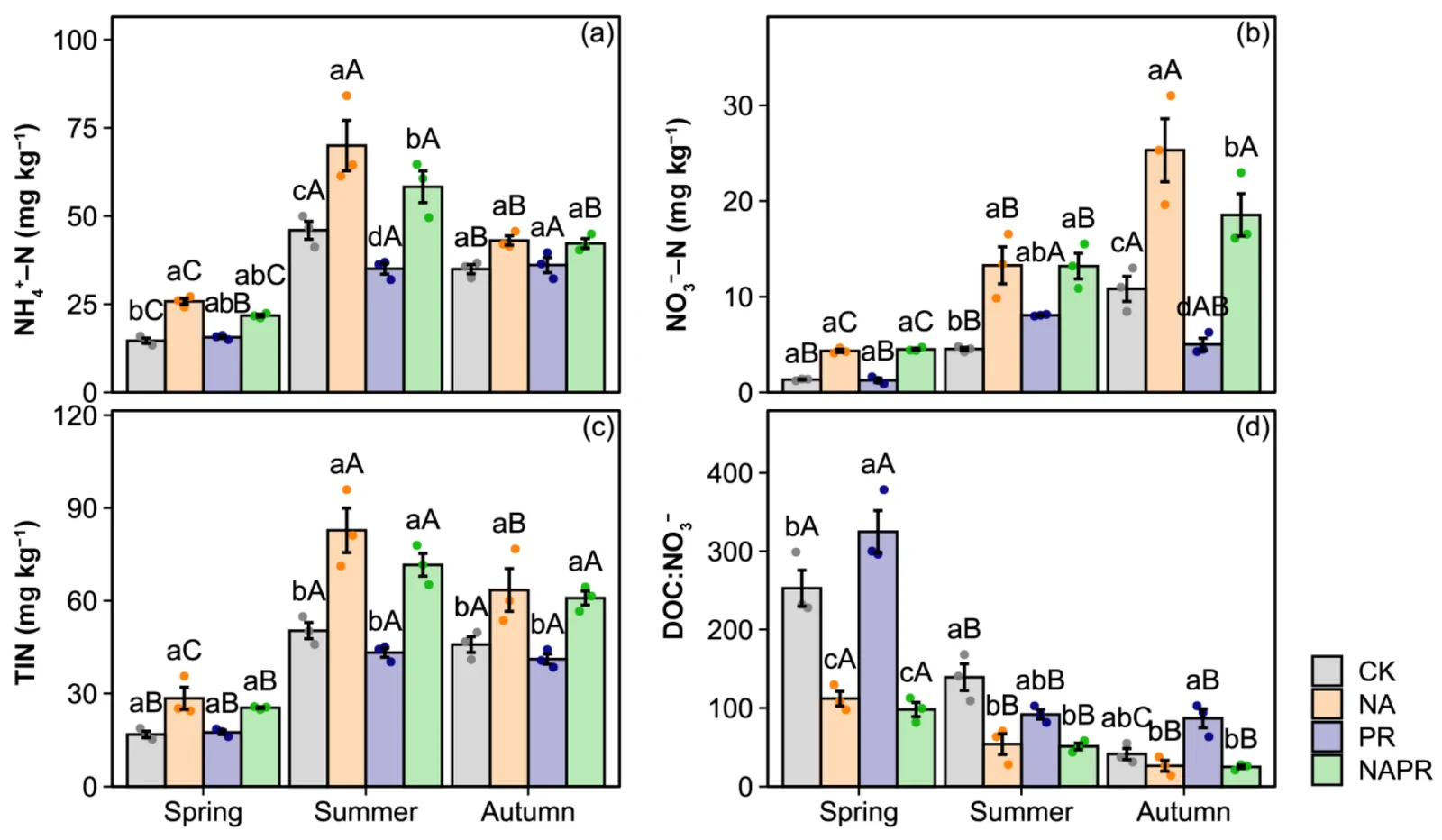
Seasonal variation in soil inorganic N availability and DOC:NO_3_^−^ ratio under nitrogen addition and reduced precipitation. (**a**) NH_4_^+^–N; (**b**) NO_3_^−^–N; (**c**) TIN; (**d**) DOC:NO_3_^−^. Bars represent means ± SE (*n* = 3), and points show individual subplot observations. Different lowercase letters indicate significant differences among treatments within the same season, whereas different uppercase letters indicate significant differences among seasons within the same treatment (Tukey-adjusted comparisons, *p* < 0.05).

Soil inorganic N availability and DOC-related stoichiometric relationships responded differentially to season, treatment, and their interaction, although response patterns varied among indicators (Figure 3; Table 4; Tables S4 and S5). Overall, NH_4_^+^–N, NO_3_^−^–N, and TIN were lower in spring and higher in summer and autumn. N addition substantially increased inorganic N availability under both precipitation regimes. Relative to the corresponding non-N treatments, mean TIN increased by 38.5–69.5% under ambient precipitation and by 45.9–65.7% under reduced precipitation across the three sampling seasons. The response of NO_3_^−^–N was particularly pronounced, whereas precipitation reduction alone had a comparatively smaller and more season-dependent effect on inorganic N availability. NO_2_^−^–N was influenced mainly by season rather than treatment.

DOC-related stoichiometric ratios also varied among treatment combinations (Figure 3d; Table S5). DOC:NO_3_^−^ showed the most pronounced treatment response, remaining generally higher under CK and PR and lower under NA and NAPR (Figure 3d). Relative to the corresponding non-N treatments, mean DOC:NO_3_^−^ decreased by 36.3–61.4% under ambient precipitation and by 44.6–71.4% under reduced precipitation across seasons. DOC:NH_4_^+^ showed a similar treatment-dependent pattern and was relatively high under PR, especially in summer (Table S5). In contrast, DOC:DON varied mainly with season, with the highest values in spring and the lowest values in autumn (Table S5). Overall, N addition increased soil inorganic N availability, whereas DOC:inorganic-N ratios generally declined under N addition, indicating a shift in dissolved C–N stoichiometry.

### 3.4. Responses of Enzyme-Related Indicators Associated with N Transformation

N-transformation-related enzyme indicators showed distinct seasonal and treatment responses, although the magnitude and direction of these responses differed among indicators (Table 5; Tables S2 and S6). AMO, NXR, NOR, and N_2_OR generally showed relatively high values under CK and lower values under PR, whereas N-added treatments tended to show intermediate responses. These indicators also exhibited pronounced seasonal variation, with AMO, NXR, NOR, and N_2_OR generally reaching higher values in summer than in spring. In contrast, NIR showed a different response pattern and was consistently highest under NAPR within each sampling season, while UE and NR displayed their own treatment-dependent responses. For example, model-estimated AMO declined from 20.62 to 15.47 U l^−1^ under CK versus PR in spring and from 46.16 to 40.60 U l^−1^ in summer, whereas NIR was highest under NAPR in all three seasons (43.61, 39.89, and 35.75 μmol d^−1^ g^−1^ in spring, summer, and autumn, respectively). Overall, the seven enzyme-related indicators did not respond uniformly to nitrogen addition and reduced precipitation, indicating process-specific responses across seasons.

Pooled relationships across all treatment–season observations suggested apparent associations between several enzyme-related indicators and ln-transformed N_2_O flux (Figure S2). However, these relationships weakened after accounting for season and repeated observations within plots. After accounting for season and repeated observations within plots, none of the seven enzyme-related indicators showed a statistically supported association with N_2_O flux (Table S8). AMO and N_2_OR, in particular, had standardized coefficients of −1.053 (95% CI: −3.158 to 1.052) and −0.106 (95% CI: −0.619 to 0.406), respectively, whereas NIR showed the strongest residual negative association (β = −0.557, 95% CI: −1.129 to 0.015). Because all of these confidence intervals included zero, the pooled enzyme–N_2_O relationships should be interpreted as season-dependent associations rather than evidence of direct process control.

### 3.5. Multivariate Associations Among Soil Properties, DOC Stoichiometry, Enzyme-Related Indicators, and N_2_O Flux

**Figure 4:**
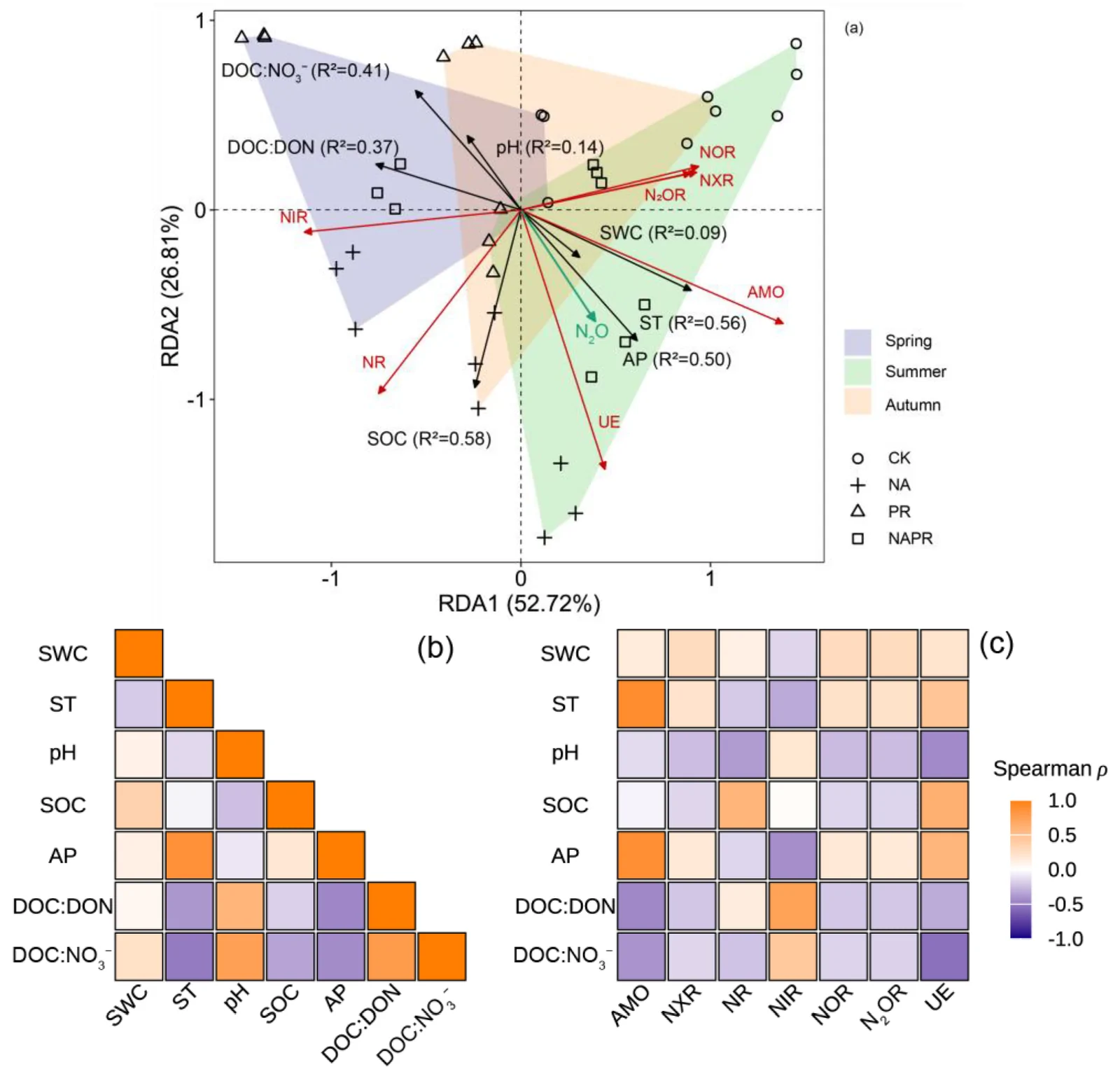
Relationships among soil environmental and resource variables, N-transformation-related enzyme indicators, and soil N_2_O flux. (**a**) Redundancy analysis (RDA) of soil environmental/resource variables and N-transformation-related enzyme indicators, with N_2_O flux fitted as a supplementary variable. (**b**) Spearman correlations among soil environmental and resource variables. (**c**) Spearman correlations between soil environmental and resource variables and N-transformation-related enzyme indicators.

**Figure 5:**
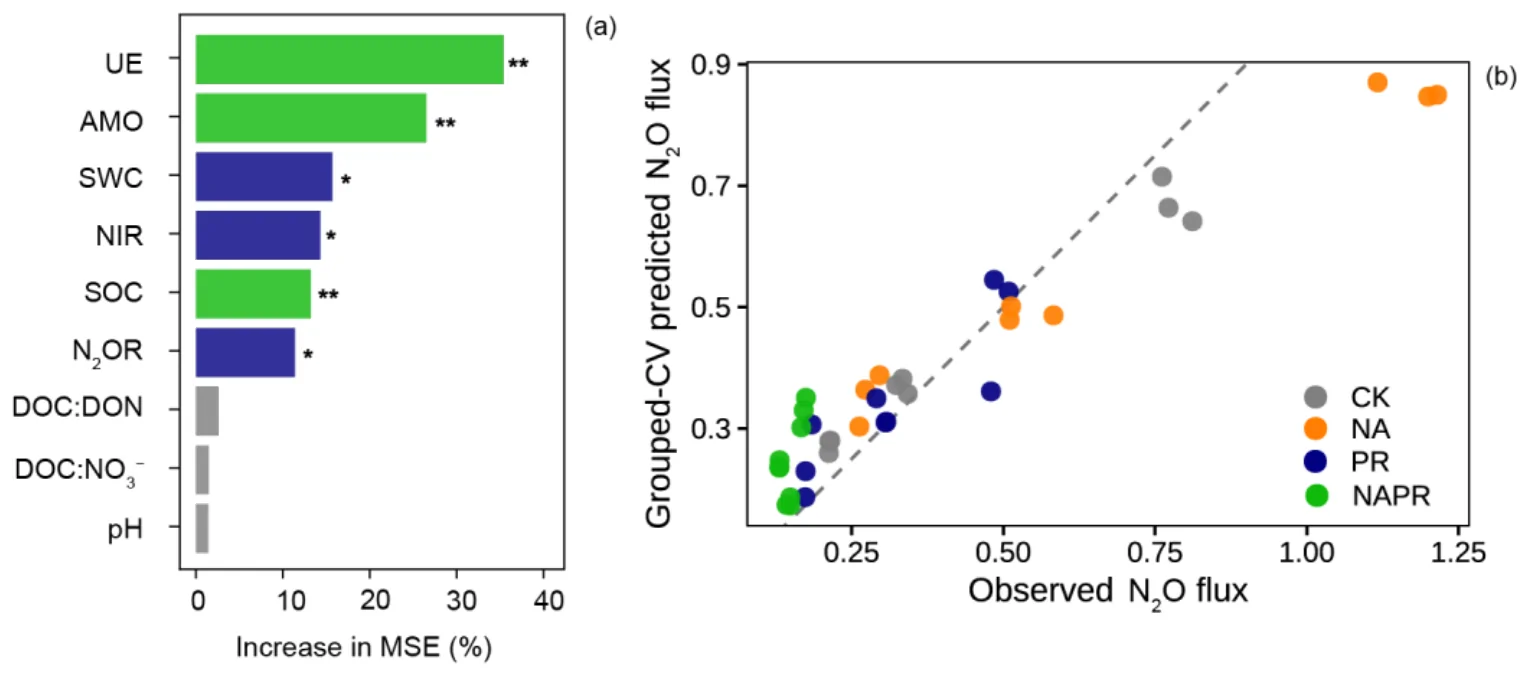
Random forest variable importance and grouped cross-validation of soil N_2_O flux. (**a**) Random forest predictor importance expressed as the percentage increase in mean squared error (Increase in MSE, %); * and ** indicate permutation-based significance at *p* < 0.05 and *p* < 0.01, respectively. (**b**) Observed versus predicted soil N_2_O flux under leave-one-main-plot-out cross-validation; the dashed line indicates the 1:1 relationship.

The multivariate analyses were broadly consistent with the univariate patterns described above (Figure 4 and Figure 5). RDA showed that the retained seven environmental variables (SWC, ST, pH, SOC, AP, DOC:DON, and DOC:NO_3_^−^) explained 59.69% of the variation in the enzyme-related indicator matrix (adjusted R^2^ = 0.496; Table S7). The first two axes together explained 79.53% of the constrained variance, with RDA1 and RDA2 accounting for 52.72% and 26.81%, respectively. ST, SOC, AP, DOC:NO_3_^−^, and DOC:DON showed comparatively stronger fits to the ordination structure, whereas the remaining variables showed weaker associations (Table S7). When fitted as a supplementary vector, monthly mean N_2_O flux projected toward the positive RDA1 and negative RDA2 directions and was broadly aligned with ST, AP, and AMO, while showing an opposite orientation to DOC:NO_3_^−^, DOC:DON, NIR, and NR (Figure 4a). Sample ordination showed clearer separation by season than by treatment, with summer samples tending to separate from spring and autumn along the main ordination space (Figure 4a).

The Spearman correlation patterns complemented the RDA results (Figure 4b,c). Among the environmental and resource variables, ST and AP covaried positively, whereas DOC:DON and DOC:NO_3_^−^ were positively associated with each other and tended to show contrasting relationships with ST and AP. Across the enzyme-related indicators, ST and AP were positively associated with AMO, whereas DOC:DON and DOC:NO_3_^−^ were negatively associated with AMO but positively associated with NIR. These patterns further indicate that variation in N-transformation-related indicators occurred within coordinated environmental and resource gradients rather than in isolation.

Random forest analysis further identified UE and AMO as the highest-ranked predictors of monthly mean soil N_2_O flux, followed by SWC, NIR, SOC, and N_2_OR (Figure 5a). Grouped cross-validation indicated good predictive performance. Predictive performance was similar across the model-internal and grouped-validation approaches, with R^2^ values ranging from 0.828 to 0.837 (Table S9). Under the primary leave-one-main-plot-out validation, the model achieved an R^2^ of 0.828, with RMSE = 0.124 and MAE = 0.091 (Figure 5b; Table S9). These results indicate that the selected soil and enzyme-related variables contained substantial predictive information for N_2_O variation across the observed treatment–season gradients, although their random-forest importance should be interpreted as predictive association rather than evidence of independent or causal effects.

## 4. Discussion

### 4.1. Reduced Precipitation Reversed Soil N_2_O Flux, Particularly in Summer

The clearest response emerging from the long-term field experiment was that precipitation regime altered the direction of the N-addition effect on soil N_2_O flux. Under ambient precipitation, N addition consistently increased N_2_O flux relative to CK, whereas under reduced precipitation, N addition further decreased N_2_O flux relative to PR, resulting in the consistent treatment order NA > CK > PR > NAPR across the three monitoring periods (Figure 2). Thus, precipitation reduction not only suppressed soil N_2_O flux but also reversed the stimulatory response to N addition.

This response coincided with consistently lower SWC under PR and NAPR across the three monitoring periods (Table 3), suggesting that altered soil moisture conditions were an important environmental context for the observed decline in N_2_O flux. Soil moisture can strongly influence N_2_O production and consumption by regulating O_2_ diffusion, redox conditions, microbial activity, and substrate transport, thereby altering the relative contributions of nitrification and denitrification to net N_2_O flux [36,37]. The suppression of N_2_O under reduced precipitation was therefore consistent with our general expectation, whereas the additional decline from PR to NAPR was not anticipated and requires separate consideration.

Long-term N addition did not offset the PR-associated decline in N_2_O flux, even though inorganic N availability increased substantially. Under NAPR, NH_4_^+^–N, NO_3_^−^–N, and TIN were all higher than under PR, despite N_2_O flux being consistently lower than under PR (Figure 2 and Figure 3a–c). Thus, additional inorganic N was available but did not translate into greater net N_2_O release under reduced precipitation. Lower soil water availability may restrict microbial activity and substrate diffusion and alter the occurrence of anaerobic microsites, thereby modifying the conditions for microbially mediated N transformations [11].

Throughfall exclusion may also have reduced throughfall-borne atmospheric N inputs to the forest floor. In forest ecosystems, throughfall N reflects not only precipitation-derived N but also dry-deposited reactive N subsequently washed from the canopy and N modified through canopy exchange processes [38]. Earlier monitoring at the Changbai Mountain Research Station showed that wet N deposition was substantial and strongly seasonal, with most annual wet N input occurring during the growing season [39]. However, throughfall chemistry and concurrent wet and dry N deposition were not measured in our experimental plots in 2023, preventing quantitative assessment of how much atmospheric N delivery was altered by the precipitation-reduction treatment. Because PR and NAPR experienced the same throughfall-reduction treatment while inorganic N availability remained higher under NAPR, altered atmospheric N delivery alone is unlikely to explain the additional decline in N_2_O flux from PR to NAPR.

The present treatment response contrasts with the earlier N_2_O pattern reported from the same long-term experimental system. During continuous measurements from November 2012 to October 2014, Geng et al. (2017) found that a 30% rainfall reduction decreased soil N_2_O emissions by 17–45%, whereas its combination with N addition increased N_2_O emissions by 58–140% [28]. Geng et al. proposed that rainfall reduction could reduce leaching of added N, allowing more N to remain in the soil and thereby enhancing N_2_O production under the combined treatment. Subsequent measurements from the same experimental system showed that throughfall reduction diminished the N-addition-induced increase in soil N leaching after six years of treatment [40], supporting an important role of water availability in regulating N retention and loss pathways.

In the present study, however, inorganic N availability remained high under NAPR while N_2_O flux was consistently the lowest. Thus, retention or accumulation of mineral N alone was insufficient to sustain high N_2_O flux after long-term combined treatment. The contrast between the earlier and present responses suggests that the relationship between N availability and net N_2_O release changed during the course of the long-term manipulation. However, this difference cannot be attributed to treatment duration alone, because interannual climatic variability, antecedent precipitation, seasonal conditions, and methodological differences may also contribute. More broadly, long-term N enrichment can progressively alter microbial N constraints and N-cycle functional capacity, such that greater N availability does not necessarily produce a sustained increase in N_2_O emissions [41].

Previous studies conducted during the course of this long-term experimental system provide site-specific evidence that prolonged N and water manipulations can affect ecosystem components not represented by instantaneous soil moisture and mineral-N measurements. Water reduction and N addition decreased fine-root length density, root biomass, and fungal hyphal length density, with particularly low values under the combined treatment [42]. Long-term N addition and reduced precipitation also altered biomass allocation and fine-root traits of dominant early-spring understory herbs in this forest [33], demonstrating that treatment effects on belowground resource acquisition extended beyond the tree layer. In addition, precipitation reduction and its interaction with N addition inhibited litter decomposition and altered litter N immobilization and nutrient release [43]. Long-term N addition and reduced precipitation also restructured soil fungal communities, including shifts in mycorrhizal and saprotrophic functional groups [44]. These site-specific observations demonstrate that prolonged manipulation can modify plant-derived C inputs, belowground resource acquisition, and nutrient turnover. More broadly, Deilmann et al. (2026) showed that forest-floor vegetation can influence N retention and N fluxes in temperate forest understories [45]. Such longer-term plant–soil feedbacks provide plausible ecological context for the contrasting response under NAPR, but because these components were not measured concurrently with the 2023 N_2_O observations, they cannot be identified as the direct cause of NAPR < PR.

The suppressive effect of reduced precipitation was strongest during the summer monitoring period, when monthly mean N_2_O fluxes were highest and treatment differences were most pronounced (Figure 2). Seasonal variation further indicates that soil moisture alone cannot explain the observed N_2_O dynamics. N_2_O flux peaked strongly in summer, whereas SWC showed comparatively limited seasonal variation within the ambient treatments; in contrast, ST increased markedly from spring to summer (Table 3). Consistently, when N_2_O was fitted as a supplementary variable in the RDA, its vector was broadly aligned with ST (Figure 4a). These patterns suggest that the summer maximum reflected the combined influence of temperature, seasonal biological activity, water availability, and rainfall-related microsite dynamics rather than a simple monotonic response to SWC. In particular, antecedent soil moisture and rainfall-driven drying–rewetting can strongly modify N_2_O and N_2_ responses by altering soil aeration and the occurrence of transient anaerobic microsites [37,46,47]. Reduced precipitation may therefore have altered the frequency and persistence of such microsites during rainfall events, while the concurrent summer increase in ST suggests an additional temperature-related contribution to the pronounced seasonal response. The RDA also showed clearer separation among seasons than among treatments, indicating that seasonal environmental variation strongly structured the measured N-transformation enzyme-related indicators (Figure 4a). This interpretation is broadly consistent with evidence that drought and rewetting can alter denitrification-derived N_2_O pulses and the seasonal importance of different N-transformation pathways [37]. Overall, reduced precipitation altered the environmental context in which added N was processed, with the strongest treatment divergence occurring in summer, but the specific process responsible for the additional suppression of N_2_O under NAPR remains unresolved. These findings support the predicted suppression of N_2_O under reduced precipitation and the expectation that N addition would not compensate for this decline; however, the further decrease from PR to NAPR was not anticipated and represents an additional long-term treatment interaction beyond our original hypothesis.

### 4.2. Context-Dependent Roles of Inorganic N Availability and DOC Stoichiometry in N_2_O Flux

The contrasting N_2_O response occurred despite substantial accumulation of inorganic N under N addition, indicating that mineral-N availability alone was insufficient to determine net N_2_O flux. Across seasons, N addition increased NH_4_^+^–N, NO_3_^−^–N, and TIN under both precipitation regimes, while DOC:NO_3_^−^ and DOC:NH_4_^+^ generally decreased (Figure 3; Tables S4 and S5). N enrichment therefore changed not only the absolute supply of inorganic N but also the balance between dissolved organic C and inorganic N. This distinction is important because ecosystem responses to chronic N enrichment depend on broader resource and environmental constraints rather than on mineral-N accumulation alone [18,48]. Previous studies have shown that dissolved C–N stoichiometry can provide important information on the coupling between C and N availability and N-cycling functions beyond that represented by bulk soil C:N alone [18]. Likewise, long-term N enrichment can reorganize dissolved C and N pools toward a more N-enriched stoichiometric environment, potentially increasing the relative importance of labile C availability for subsequent N transformations [13,16,49,50].

Against this background, treatment effects were expressed not only as changes in inorganic N availability but also as shifts in dissolved C–inorganic N ratios. DOC:NO_3_^−^ showed the clearest treatment response, remaining generally higher under CK and PR and lower under NA and NAPR, whereas DOC:NH_4_^+^ showed a broadly similar treatment-dependent pattern and was relatively high under PR, especially in summer (Figure 3d; Table S5). These patterns indicate that N addition altered the relative availability of dissolved organic C and inorganic N rather than simply increasing N substrate supply [18,27]. Consistent with this interpretation, higher NH_4_^+^–N, NO_3_^−^–N, and TIN under N-added treatments did not translate directly into higher N_2_O flux under reduced precipitation. Thus, N enrichment changed not only the amount of available inorganic N but also the C–N resource environment within which microbial N transformations occurred.

Site-specific evidence supports the possibility that long-term treatments also altered the turnover of plant-derived substrates. In a litterbag experiment using the same 30% throughfall-reduction and 50 kg N ha^−1^ yr^−1^ N-addition levels, precipitation reduction and its interaction with N addition inhibited litter decomposition, while N immobilization and nutrient release depended on litter identity [43]. This result does not demonstrate that litter processes caused the N_2_O response observed in 2023, but it shows that the same long-term manipulations can modify C and nutrient turnover before plant-derived substrates enter soil dissolved pools. Together with the observed shifts in DOC stoichiometry, these findings support a broader resource-context interpretation rather than one based solely on mineral-N availability.

Evidence from other experimental systems further illustrates why increased NO_3_^−^ availability need not produce a proportional increase in net N_2_O release. DOC:NO_3_^−^ stoichiometry has been shown experimentally to regulate N_2_O responses [22], while isotope-pool-dilution measurements demonstrated that additions of organic C can increase gross N_2_O consumption and modify net N_2_O emissions across soils [19]. Direct measurements of both N_2_O and N_2_ further showed that the effect of NO_3_^−^ availability on the denitrification product ratio depends strongly on soil pH [23], while sustained labile-C inputs from artificial roots can alter the relative production of N_2_O and N_2_ [24]. Although these systems differ from the present forest, together they demonstrate that net N_2_O flux depends on interacting C, N, pH, moisture, and redox conditions rather than on nitrate availability alone.

DOC-related stoichiometric relationships also varied seasonally. DOC:DON showed a particularly clear seasonal pattern, whereas DOC:NO_3_^−^ and DOC:NH_4_^+^ varied with both season and treatment (Figure 3d; Table S5). Such variation indicates that the relevance of dissolved C–N stoichiometry is itself season-dependent and embedded within changing environmental conditions. DOC stoichiometry should therefore be interpreted here as part of the environmental and substrate context associated with N transformation, rather than as a direct determinant of N_2_O flux.

Collectively, the multivariate analyses support this context-dependent interpretation. In the RDA and correlation analyses, DOC:DON and DOC:NO_3_^−^ gradients covaried with several N-transformation-related enzyme indicators (Figure 4), indicating that dissolved C–N stoichiometry formed part of the broader resource environment associated with N transformation. These relationships are consistent with the view that C–N resource balance can influence the relative conditions for N_2_O-producing and N_2_O-consuming processes [19,22,23,24,51]. Thus, DOC stoichiometry is best interpreted here as an indicator of the substrate context in which N-transformation processes varied, rather than as an independent determinant of N_2_O flux. These results support our second hypothesis that inorganic N availability and DOC stoichiometry were associated with N_2_O within a broader environmental and resource context rather than acting as dominant standalone controls.

### 4.3. Context-Dependent Associations Between N-Transformation-Related Indicators and Soil N_2_O Flux

The N-transformation-related enzyme indicators provided information on the process context of N_2_O variation, but their treatment responses did not directly mirror the pattern of net N_2_O flux. Under ambient precipitation, N addition increased N_2_O flux relative to CK, whereas AMO and N_2_OR were generally lower under NA than under CK (Figure 2; Table 5). Under reduced precipitation, N addition further decreased N_2_O flux from PR to NAPR, while both AMO and N_2_OR were generally higher under NAPR than under PR. Thus, neither indicator showed a simple monotonic correspondence with the treatment response of N_2_O. This divergence is important because net soil N_2_O flux reflects the balance among multiple N_2_O-producing and N_2_O-consuming processes rather than the activity of a single transformation step [19,26,52,53].

AMO is associated with the initial oxidation of NH_4_^+^ during nitrification and therefore represents an indicator of nitrification-related conditions potentially contributing to N_2_O production. Ammonia oxidation is an important entry step in nitrification-associated N_2_O formation and is sensitive to NH_4_^+^ availability, pH, and soil water conditions [20,25,54,55]. In the present study, AMO showed pronounced seasonal variation and ranked among the highest predictors in the random-forest analysis (Figure 5a). However, the apparent pooled AMO–N_2_O relationship weakened after season and repeated observations within plots were accounted for, and the season-adjusted association was not statistically supported (Figure S2; Table S8). The high RF importance of AMO therefore most likely reflects its predictive information across the combined treatment–season environmental gradient rather than evidence that AMO independently controlled N_2_O flux.

N_2_OR represents an indicator associated with the terminal reduction of N_2_O to N_2_ and therefore relates to the sink side of the soil N_2_O budget. Biological N_2_O reduction can strongly influence the fraction of denitrified N ultimately emitted as N_2_O [19,26]. However, the N_2_OR measurement used here is an enzyme-related indicator rather than a direct measurement of the N_2_O-to-N_2_ reduction rate. Its treatment pattern did not correspond directly to N_2_O flux, and its season-adjusted association with N_2_O was also not statistically supported (Table S8). Consequently, the particularly low N_2_O flux under NAPR cannot be attributed to enhanced terminal N_2_O reduction on the basis of the present data. Direct measurements of N_2_ production or gross N_2_O production and consumption would be required to distinguish changes in N_2_O sources from changes in N_2_O sinks [19,23,51].

The responses of the remaining indicators further support a process-specific rather than uniform adjustment of soil N transformation. NIR was consistently highest under NAPR, whereas NXR and NOR broadly followed the treatment patterns of AMO and N_2_OR, and UE and NR responded differently (Table 4; Table S6). This heterogeneous response is consistent with previous work in the same broad-leaved Korean pine forest, where long-term N addition, rainfall reduction, and their combination differentially altered net nitrification and nitrifying and denitrifying functional groups, while denitrification also showed pronounced seasonal variation [56]. Experimental studies from other ecosystems similarly show that changes in precipitation and drying–rewetting conditions can affect nitrification, denitrification, and N_2_O consumption to different degrees [37,57,58]. More broadly, soil physicochemical conditions can also structure denitrifying bacterial communities and are associated with differences in N_2_O accumulation patterns [59]. Together with the RDA and correlation patterns in the present study (Figure 4), these results indicate that N_2_O variation was embedded within coordinated but non-uniform changes in soil environment, resource availability, and N-transformation-related processes. The enzyme-related indicators therefore provide useful process context, but they do not identify a single pathway responsible for the additional suppression of N_2_O under NAPR.

Because the enzyme measurements were process-related indicators rather than direct transformation rates, and N_2_ production was not measured, the relative contributions of altered N_2_O production and consumption cannot be resolved from the present dataset. The results should therefore be interpreted as evidence of context-dependent coordination among environmental conditions and N-transformation-related indicators rather than as proof of a specific causal pathway. These findings support our third hypothesis that N-transformation-related indicators respond in a season-dependent manner and that their relationships with net N_2_O flux vary across the treatment–season environmental context, rather than reflecting fixed or uniform process controls.

## 5. Conclusions

Reduced precipitation consistently decreased soil N_2_O flux across the three monitoring periods in 2023 in this temperate forest and reversed the stimulatory response to N addition observed under ambient precipitation, resulting in the consistent treatment order NA > CK > PR > NAPR, with the strongest treatment divergence occurring during the summer monitoring period. Although long-term N addition substantially increased inorganic N availability, it did not restore N_2_O flux under reduced precipitation, and N_2_O flux declined further from PR to NAPR, indicating that increased N substrate availability alone was insufficient to sustain higher net N_2_O release under reduced precipitation. Multivariate analyses showed that soil environmental and resource gradients covaried with N-transformation-related indicators, while random forest analysis identified UE and AMO among the highest-ranked predictors of N_2_O variation and retained good predictive performance under grouped cross-validation. However, none of the measured enzyme-related indicators showed a statistically supported season-adjusted association with N_2_O flux, and their treatment responses did not directly mirror the pattern of net N_2_O flux, indicating that these relationships should be interpreted as context-dependent covariation and predictive information rather than direct evidence of specific production or reduction pathways. Overall, these findings emphasize that soil N_2_O responses to long-term N enrichment under reduced precipitation depend not only on inorganic N availability but also on water availability, C–N resource conditions, soil environmental context, and multiple N-transformation-related responses. The particularly low N_2_O flux under NAPR could not be attributed to a single measured pathway. Future studies integrating year-round N_2_O monitoring with direct measurements of N_2_O production, N_2_O consumption, and N_2_ production will help clarify the processes underlying this long-term treatment interaction.

## Figures and Tables

**Table 1:** Basic characteristics of the surface soil at the study site.

Soil Layer	Sampling Depth (cm)	Soil Texture	TC (g/kg)	TN (g/kg)	TC:TN
Surface mineral soil	0–10	Loam	61.22	3.94	15.77

Note: TC, total carbon; TN, total nitrogen. Values are three-season means for CK plots in 2023 (*n* = 3).

**Table 2:** Model-estimated soil N_2_O flux under different treatments across sampling seasons.

Season	CK	NA	PR	NAPR
Spring	0.214 (0.204, 0.224)	0.277 (0.264, 0.290)	0.177 (0.169, 0.186)	0.147 (0.140, 0.154)
Summer	0.781 (0.746, 0.819)	1.176 (1.123, 1.232)	0.491 (0.468, 0.514)	0.171 (0.163, 0.179)
Autumn	0.333 (0.318, 0.349)	0.534 (0.510, 0.560)	0.301 (0.288, 0.316)	0.131 (0.125, 0.137)

Note: Values are back-transformed estimated marginal means with 95% confidence intervals from the hierarchical linear mixed-effects model fitted to ln-transformed N_2_O flux. Estimates are presented on the original N_2_O flux scale (nmol m^−2^ s^−1^).

**Table 3:** Seasonal variation in selected soil physicochemical properties across treatments.

Treatment	ST (°C)	SWC (%)	pH	SOC (mg/g)	AP (mg/kg)
Spring					
CK	7.25 ± 0.06 cC	44.21 ± 0.841 bA	5.37 ± 0.01 abA	60.97 ± 0.98 abA	13.11 ± 0.26 aC
NA	7.22 ± 0.13 cC	47.56 ± 0.99 aA	5.17 ± 0.05 cA	67.91 ± 1.70 aA	14.90 ± 0.18 aC
PR	7.69 ± 0.18 bC	36.15 ± 0.97 cAB	5.40 ± 0.01 aA	52.36 ± 1.48 cB	11.53 ± 0.27 aC
NAPR	8.79 ± 0.04 aC	33.30 ± 0.20 cB	5.23 ± 0.03 bcA	53.48 ± 1.63 bcA	13.47 ± 1.14 aC
Summer					
CK	16.65 ± 0.03 cA	44.97 ± 0.57 aA	5.30 ± 0.01 abA	49.23 ± 1.29 cB	37.31 ± 2.16 bA
NA	16.67 ± 0.03 cA	43.27 ± 0.46 aB	5.23 ± 0.08 bcA	68.25 ± 0.63 aA	51.38 ± 1.71 aA
PR	17.21 ± 0.02 bA	38.44 ± 0.78 bA	5.45 ± 0.02 aA	61.99 ± 4.41 abA	42.71 ± 1.10 bA
NAPR	17.80 ± 0.04 aA	35.98 ± 0.78 bA	5.09 ± 0.04 cB	57.75 ± 2.18 bA	29.94 ± 2.57 cA
Autumn					
CK	14.58 ± 0.05 bB	44.75 ± 0.71 aA	5.14 ± 0.02 aB	57.52 ± 0.37 bA	19.42 ± 1.05 bB
NA	14.60 ± 0.10 bB	41.91 ± 0.90 aB	4.95 ± 0.04 bB	73.75 ± 2.82 aA	25.65 ± 2.34 aB
PR	13.69 ± 0.05 cB	35.79 ± 1.00 bB	5.25 ± 0.01 aB	54.74 ± 2.39 bAB	20.75 ± 1.67 abB
NAPR	15.46 ± 0.08 aB	30.54 ± 0.61 cC	4.94 ± 0.07 bC	55.50 ± 1.38 bA	21.56 ± 1.34 abB

Note: Values are means ± SE (*n* = 3). Different lowercase letters within the same season indicate significant differences among treatments, whereas different uppercase letters within the same treatment indicate significant differences among seasons, based on estimated marginal means with Tukey adjustment (*p* < 0.05). ST, soil temperature; SWC, soil water content; SOC, soil organic carbon; AP, available phosphorus.

**Table 4:** Fixed effects of nitrogen addition, precipitation reduction, and season on soil inorganic N availability and DOC:NO_3_^−^ ratio.

Effect	NH_4_^+^–N		NO_3_^−^–N		TIN		DOC:NO_3_^−^	
	F (df1, df2)	*p*	F (df1, df2)	*p*	F (df1, df2)	*p*	F (df1, df2)	*p*
NA	83.82 (1, 4)	<0.001	179.18 (1, 4)	<0.001	51.46 (1, 4)	0.002	4643.40 (1, 4)	<0.001
PR	10.11 (1, 4)	0.034	2.33 (1, 4)	0.202	6.64 (1, 4)	0.061	0.67 (1, 4)	0.458
Season	127.68 (2, 16)	<0.001	75.51 (2, 16)	<0.001	162.75 (2, 16)	<0.001	133.08 (2, 16)	<0.001
NA × PR	0.81 (1, 4)	0.418	1.45 (1, 4)	0.294	0.12 (1, 4)	0.748	111.71 (1, 4)	<0.001
NA × Season	9.71 (2, 16)	0.002	15.75 (2, 16)	<0.001	9.90 (2, 16)	0.002	32.30 (2, 16)	<0.001
PR × Season	4.46 (2, 16)	0.029	9.19 (2, 16)	0.002	1.53 (2, 16)	0.246	4.64 (2, 16)	0.026
NA × PR × Season	0.14 (2, 16)	0.871	0.50 (2, 16)	0.616	0.27 (2, 16)	0.764	6.00 (2, 16)	0.011

Note: F and *p* values are from design-based split-plot tests for the fixed effects of nitrogen addition (NA), precipitation reduction (PR), season, and their interactions. Numerator and denominator degrees of freedom are shown in parentheses after F.

**Table 5:** Model-estimated means of N-transformation-related enzyme indicators under nitrogen addition and reduced precipitation across sampling seasons.

Treatment	UE μg/d/g	AMO (U/l)	NXR (U/l)	NR μmol/d/g	NIR μmol/d/g	NOR (U/l)	N_2_OR (U/l)
Spring							
CK	304.28 (291.07, 317.49) ab	20.62 (19.74, 21.50) a	57.92 (57.04, 58.79) a	0.63 (0.62, 0.64) bc	39.62 (38.48, 40.76) b	260.29 (259.87, 260.71) a	141.96 (141.12, 142.80) a
NA	320.34 (307.13, 333.55) a	17.93 (17.05, 18.81) b	40.23 (39.35, 41.10) c	0.65 (0.64, 0.66) ab	41.31 (40.17, 42.45) b	200.94 (200.52, 201.36) c	117.11 (116.28, 117.95) c
PR	228.11 (214.90, 241.32) c	15.47 (14.59, 16.35) c	30.22 (29.35, 31.10) d	0.62 (0.61, 0.63) c	39.61 (38.47, 40.75) b	175.22 (174.80, 175.64) d	104.34 (103.50, 105.18) d
NAPR	284.75 (271.54, 297.96) b	18.79 (17.91, 19.67) b	49.05 (48.17, 49.93) b	0.65 (0.64, 0.66) a	43.61 (42.47, 44.76) a	228.22 (227.80, 228.63) b	130.74 (129.91, 131.58) b
Summer							
CK	297.87 (284.66, 311.08) c	46.16 (45.28, 47.04) a	64.31 (63.44, 65.19) a	0.58 (0.57, 0.59) c	34.43 (33.29, 35.58) c	273.38 (272.97, 273.80) a	148.50 (147.67, 149.34) a
NA	382.30 (369.09, 395.51) a	42.82 (41.94, 43.70) b	45.87 (44.99, 46.75) c	0.65 (0.64, 0.66) a	37.84 (36.70, 38.98) ab	221.45 (221.03, 221.87) c	125.77 (124.93, 126.60) c
PR	303.99 (290.78, 317.20) c	40.60 (39.72, 41.48) c	36.87 (35.99, 37.75) d	0.61 (0.60, 0.62) b	36.02 (34.88, 37.16) bc	191.23 (190.81, 191.64) d	112.36 (111.53, 113.20) d
NAPR	334.17 (320.96, 347.37) b	44.52 (43.64, 45.40) ab	55.08 (54.21, 55.96) b	0.64 (0.63, 0.65) a	39.89 (38.75, 41.04) a	249.66 (249.24, 250.08) b	138.39 (137.56, 139.23) b
Autumn							
CK	292.93 (279.72, 306.13) b	32.55 (31.67, 33.43) a	59.93 (59.06, 60.81) a	0.62 (0.61, 0.63) b	30.20 (29.06, 31.35) c	267.81 (267.39, 268.23) a	144.74 (143.90, 145.58) a
NA	325.47 (312.26, 338.68) a	28.70 (27.82, 29.58) c	43.13 (42.25, 44.00) c	0.67 (0.66, 0.68) a	32.87 (31.72, 34.01) b	214.08 (213.66, 214.50) c	122.01 (121.18, 122.85) c
PR	261.99 (248.78, 275.20) c	26.81 (25.93, 27.69) d	33.39 (32.51, 34.27) d	0.59 (0.58, 0.60) c	30.50 (29.36, 31.64) c	185.58 (185.16, 186.00) d	108.95 (108.12, 109.79) d
NAPR	315.54 (302.33, 328.74) ab	30.45 (29.57, 31.33) b	52.24 (51.36, 53.11) b	0.61 (0.60, 0.62) b	35.75 (34.61, 36.89) a	242.59 (242.17, 243.01) b	135.07 (134.23, 135.91) b

Note: Values are estimated marginal means with 95% confidence intervals from the linear mixed-effects models. Different lowercase letters indicate significant differences among treatments within the same sampling season (Tukey-adjusted comparisons, *p* < 0.05). CK, control; NA, nitrogen addition; PR, precipitation reduction; NAPR, combined nitrogen addition and precipitation reduction.

## Data Availability

Data will be made available on request.

## Supplementary Materials

The following supporting information can be downloaded at: https://www.mdpi.com/article/10.3390/plants15213436/s1, Table S1: Effects of nitrogen addition on soil N_2_O flux under ambient and reduced precipitation; Table S2: LMM fixed tests of nitrogen addition, precipitation reduction, season, and their interactions on soil N_2_O flux and related soil variables; Table S3: Seasonal variation in soil physicochemical properties and elemental stoichiometric ratios across treatments; Table S4: Seasonal variation in soil inorganic nitrogen availability and related stoichiometric ratios across treatments; Table S5: Seasonal variation in dissolved organic matter, available phosphorus, and DOC-based stoichiometric ratios across treatments; Table S6: Seasonal variation in N-transformation-related enzyme indicators across treatments; Table S7: Summary statistics and diagnostics for the redundancy analysis; Table S8: Season-adjusted associations between N-transformation-related enzyme indicators and soil N_2_O flux; Table S9: Predictive performance of the random-forest model under out-of-bag and grouped cross-validation; Figure S1: Daily environmental conditions and soil N_2_O flux dynamics during the three measurement periods; Figure S2: Pooled relationships between soil N-transformation-related enzyme indicators and ln-transformed N_2_O flux.

## Abbreviations

The following abbreviations are used in this manuscript:

CK	natural precipitation control
NA	nitrogen addition
PR	precipitation reduction
NAPR	nitrogen addition combined with precipitation reduction
