# Supplementary material for: Long-Term Nitrogen Addition and Reduced Precipitation Alter Seasonal Soil N2O Fluxes and N-Transformation Processes in a Temperate Forest
Source: Plants (Basel). 2026 Nov 4;15(21):3436. doi: 10.3390/plants15213436 (PMC13765729; doi:10.3390/plants15213436)
Supplement: Supplementary file 1 [file plants-15-03436-s001.zip › plants-4531769-supplementary.pdf]

Supplementary Materials

Table S1. Effects of nitrogen addition on soil N_2O flux under ambient and reduced precipitation.

Contrast	Absolute effect (nmol m^{-2} s^{-1}; 95% CI)	Relative effect (%, 95% CI)	*p*
**Spring**			
NA - CK	0.0632 (0.0494, 0.0770)	29.6 (22.7, 36.9)	<0.001
NAPR - PR	-0.0304 (-0.0394, -0.0213)	-17.1 (-21.6, -12.4)	<0.001
PR - CK	-0.0364 (-0.0493, -0.0234)	-17.0 (-22.3, -11.4)	<0.001
NAPR - NA	-0.1299 (-0.1446, -0.1153)	-46.9 (-50.3, -43.3)	<0.001
NAPR - CK	-0.0667 (-0.0788, -0.0546)	-31.2 (-35.6, -26.5)	<0.001
(NAPR - PR) - (NA - CK)	-0.0936 (-0.1101, -0.0771)	-36.1 (-40.8, -30.9)	<0.001
**Summer**			
NA - CK	0.3948 (0.3387, 0.4510)	50.5 (42.5, 59.0)	<0.001
NAPR - PR	-0.3199 (-0.3418, -0.2979)	-65.2 (-67.0, -63.2)	<0.001
PR - CK	-0.2905 (-0.3336, -0.2474)	-37.2 (-41.2, -32.9)	<0.001
NAPR - NA	-1.0052 (-1.0607, -0.9497)	-85.5 (-86.4, -84.5)	<0.001
NAPR - CK	-0.6103 (-0.6477, -0.5730)	-78.1 (-79.5, -76.6)	<0.001
(NAPR - PR) - (NA - CK)	-0.7147 (-0.7749, -0.6544)	-76.9 (-78.6, -75.0)	<0.001
**Autumn**			
NA - CK	0.2010 (0.1758, 0.2261)	60.3 (51.7, 69.4)	<0.001
NAPR - PR	-0.1701 (-0.1837, -0.1566)	-56.5 (-58.8, -54.0)	<0.001
PR - CK	-0.0320 (-0.0530, -0.0110)	-9.6 (-15.4, -3.4)	0.005
NAPR - NA	-0.4031 (-0.4288, -0.3774)	-75.5 (-77.0, -73.8)	<0.001
NAPR - CK	-0.2021 (-0.2188, -0.1854)	-60.6 (-63.2, -58.0)	<0.001
(NAPR - PR) - (NA - CK)	-0.3711 (-0.3997, -0.3425)	-72.8 (-74.9, -70.6)	<0.001
**Total**			
NA - CK	0.2197 (0.1979, 0.2415)	46.2 (40.1, 52.7)	<0.001
NAPR - PR	-0.1735 (-0.1833, -0.1636)	-49.9 (-52.0, -47.7)	<0.001
PR - CK	-0.1196 (-0.1395, -0.0997)	-22.2 (-26.4, -17.7)	<0.001
NAPR - NA	-0.5127 (-0.5376, -0.4878)	-73.3 (-74.8, -71.8)	<0.001
NAPR - CK	-0.2931 (-0.3101, -0.2760)	-61.0 (-63.1, -58.8)	<0.001
(NAPR - PR) - (NA - CK)	-0.3931 (-0.4170, -0.3692)	-65.7 (-67.8, -63.6)	<0.001

Note: CK, NA, PR, and NAPR denote the control, nitrogen addition, precipitation reduction, and combined treatments, respectively. NA−CK and NAPR−PR represent the effects of nitrogen addition under ambient and reduced precipitation, respectively. Total represents the estimates across the three monitoring periods.

Table S2. LMM fixed tests of nitrogen addition, precipitation reduction, season, and their interactions on soil N_2O flux and related soil variables.

Indicator	NA		PR		Season		NA×PR		NA×Season		PR×Season		NA×PR×Season	
	F (df1, df2)	*p*	F (df1, df2)	*p*	F (df1, df2)	*p*	F (df1, df2)	*p*	F (df1, df2)	*p*	F (df1, df2)	*p*	F (df1, df2)	*p*

N_2O	200.61 (1, 4)	< 0.001	1505.75 (1, 4)	< 0.001	2938.91 (2, 16)	< 0.001	2376.25 (1, 4)	< 0.001	98.30 (2, 16)	< 0.001	469.48 (2, 16)	< 0.001	225.48 (2, 16)	< 0.001
ST	137.05 (1, 4)	< 0.001	222.87 (1, 4)	< 0.001	13291.89 (2, 16)	< 0.001	134.43 (1, 4)	< 0.001	12.61 (2, 16)	< 0.001	43.67 (2, 16)	< 0.001	12.58 (2, 16)	< 0.001
SWC	14.80 (1, 4)	0.018	402.69 (1, 4)	< 0.001	14.11 (2, 16)	< 0.001	9.42 (1, 4)	0.037	9.60 (2, 16)	0.002	10.29 (2, 16)	0.001	4.05 (2, 16)	0.038
pH	44.26 (1, 4)	0.003	1.26 (1, 4)	0.324	51.49 (2, 16)	< 0.001	3.82 (1, 4)	0.122	0.93 (2, 16)	0.416	0.66 (2, 16)	0.53	5.52 (2, 16)	0.015
TC	4.75 (1, 4)	0.095	6.09 (1, 4)	0.069	6.54 (2, 16)	0.008	30.85 (1, 4)	0.005	1.00 (2, 16)	0.388	0.93 (2, 16)	0.414	12.87 (2, 16)	< 0.001
TN	3.93 (1, 4)	0.118	3.34 (1, 4)	0.142	9.99 (2, 16)	0.002	9.33 (1, 4)	0.038	1.69 (2, 16)	0.215	0.65 (2, 16)	0.537	5.20 (2, 16)	0.018
TP	3.98 (1, 4)	0.117	8.09 (1, 4)	0.047	56.98 (2, 16)	< 0.001	38.53 (1, 4)	0.003	0.56 (2, 16)	0.579	7.74 (2, 16)	0.004	1.47 (2, 16)	0.26
NH_4^+	83.82 (1, 4)	< 0.001	10.11 (1, 4)	0.034	127.68 (2, 16)	< 0.001	0.81 (1, 4)	0.418	9.71 (2, 16)	0.002	4.46 (2, 16)	0.029	0.14 (2, 16)	0.871
NO_3^-	179.18 (1, 4)	< 0.001	2.33 (1, 4)	0.202	75.51 (2, 16)	< 0.001	1.45 (1, 4)	0.294	15.75 (2, 16)	< 0.001	9.19 (2, 16)	0.002	0.50 (2, 16)	0.616
NO_2^-	0.83 (1, 4)	0.414	0.46 (1, 4)	0.534	60.78 (2, 16)	< 0.001	1.22 (1, 4)	0.331	0.77 (2, 16)	0.479	1.13 (2, 16)	0.348	0.34 (2, 16)	0.716
TIN	51.46 (1, 4)	0.002	6.64 (1, 4)	0.061	162.75 (2, 16)	< 0.001	0.12 (1, 4)	0.748	9.90 (2, 16)	0.002	1.53 (2, 16)	0.246	0.27 (2, 16)	0.764
SOC	27.99 (1, 4)	0.006	32.39 (1, 4)	0.005	0.73 (2, 16)	0.497	34.99 (1, 4)	0.004	1.34 (2, 16)	0.291	12.23 (2, 16)	< 0.001	4.72 (2, 16)	0.024
DOC	18.33 (1, 4)	0.013	2.75 (1, 4)	0.173	150.50 (2, 16)	< 0.001	58.85 (1, 4)	0.002	1.70 (2, 16)	0.214	12.09 (2, 16)	< 0.001	5.07 (2, 16)	0.02
DON	65.74 (1, 4)	0.001	0.20 (1, 4)	0.68	116.83 (2, 16)	< 0.001	24.27 (1, 4)	0.008	2.60 (2, 16)	0.105	3.83 (2, 16)	0.044	1.94 (2, 16)	0.176
AP	4.34 (1, 4)	0.106	14.66 (1, 4)	0.019	361.03 (2, 16)	< 0.001	30.61 (1, 4)	0.005	0.98 (2, 16)	0.398	6.80 (2, 16)	0.007	23.91 (2, 16)	< 0.001
C:N	8.76 (1, 4)	0.042	27.34 (1, 4)	0.006	27.57 (2, 16)	< 0.001	1.68 (1, 4)	0.264	2.96 (2, 16)	0.081	1.10 (2, 16)	0.358	1.15 (2, 16)	0.342
C:P	2.43 (1, 4)	0.194	3.89 (1, 4)	0.12	38.21 (2, 16)	< 0.001	9.27 (1, 4)	0.038	0.50 (2, 16)	0.616	8.34 (2, 16)	0.003	2.16 (2, 16)	0.148
N:P	12.70 (1, 4)	0.024	0.09 (1, 4)	0.781	36.60 (2, 16)	< 0.001	6.42 (1, 4)	0.064	3.34 (2, 16)	0.061	15.17 (2, 16)	< 0.001	4.48 (2, 16)	0.029
NH_4^+: NO_3^-	445.65 (1, 4)	< 0.001	0.01 (1, 4)	0.937	23.53 (2, 16)	< 0.001	2.43 (1, 4)	0.194	4.12 (2, 16)	0.036	11.86 (2, 16)	< 0.001	11.33 (2, 16)	< 0.001
NO_3^-: NO_2^-	33.64 (1, 4)	0.004	0.23 (1, 4)	0.655	68.34 (2, 16)	< 0.001	0.04 (1, 4)	0.855	10.64 (2, 16)	0.001	0.24 (2, 16)	0.788	2.83 (2, 16)	0.088
DOC: DON	5.54 (1, 4)	0.078	3.60 (1, 4)	0.131	39.80 (2, 16)	< 0.001	1.65 (1, 4)	0.268	0.44 (2, 16)	0.65	0.60 (2, 16)	0.561	0.13 (2, 16)	0.881
DOC: AP	0.62 (1, 4)	0.475	7.39 (1, 4)	0.053	62.89 (2, 16)	< 0.001	0.30 (1, 4)	0.611	0.20 (2, 16)	0.817	2.45 (2, 16)	0.118	5.11 (2, 16)	0.019
DON: AP	0.90 (1, 4)	0.397	0.37 (1, 4)	0.577	23.69 (2, 16)	< 0.001	2.74 (1, 4)	0.173	0.37 (2, 16)	0.698	0.68 (2, 16)	0.522	3.76 (2, 16)	0.046
DOC: NO_3^-	4643.40 (1, 4)	< 0.001	0.67 (1, 4)	0.458	133.08 (2, 16)	< 0.001	111.71 (1, 4)	< 0.001	32.30 (2, 16)	< 0.001	4.64 (2, 16)	0.026	6.00 (2, 16)	0.011
DOC: NH_4^+	23.34 (1, 4)	0.008	6.50 (1, 4)	0.063	66.86 (2, 16)	< 0.001	4.05 (1, 4)	0.115	2.38 (2, 16)	0.124	4.83 (2, 16)	0.023	1.32 (2, 16)	0.294

AMO	57.92 (1, 4)	0.002	20.60 (1, 4)	0.011	8582.20 (2, 16)	< 0.001	25041.81 (1, 4)	< 0.001	0.73 (2, 16)	0.498	0.17 (2, 16)	0.844	2.10 (2, 16)	0.154
NXR	7.85 (1, 4)	0.049	849.11 (1, 4)	< 0.001	222.20 (2, 16)	< 0.001	10745.40 (1, 4)	< 0.001	1.90 (2, 16)	0.182	0.76 (2, 16)	0.486	0.43 (2, 16)	0.658
NR	258.79 (1, 4)	< 0.001	13.24 (1, 4)	0.022	14.64 (2, 16)	< 0.001	12.86 (1, 4)	0.023	8.80 (2, 16)	0.003	33.79 (2, 16)	< 0.001	7.02 (2, 16)	0.006
NIR	89.49 (1, 4)	< 0.001	25.82 (1, 4)	0.007	262.22 (2, 16)	< 0.001	5.91 (1, 4)	0.072	1.12 (2, 16)	0.352	0.41 (2, 16)	0.671	1.14 (2, 16)	0.344
NOR	21.52 (1, 4)	0.01	43101.17 (1, 4)	< 0.001	8660.44 (2, 16)	< 0.001	204702.88 (1, 4)	< 0.001	299.95 (2, 16)	< 0.001	34.85 (2, 16)	< 0.001	7.42 (2, 16)	0.005
N_2OR	43.26 (1, 4)	0.003	1261.81 (1, 4)	< 0.001	471.20 (2, 16)	< 0.001	14136.98 (1, 4)	< 0.001	2.10 (2, 16)	0.156	0.80 (2, 16)	0.468	3.94 (2, 16)	0.041
UE	61.43 (1, 4)	0.001	256.52 (1, 4)	< 0.001	65.53 (2, 16)	< 0.001	0.04 (1, 4)	0.844	3.53 (2, 16)	0.054	12.68 (2, 16)	< 0.001	19.29 (2, 16)	< 0.001

Note: Values are F statistics with numerator and denominator degrees of freedom in parentheses, followed by p values. Linear mixed-effects models were fitted as Y ~ NA × PR × Season + (1 | MainPlot) + (1 | Plot), where MainPlot represents the six precipitation whole plots (precipitation reduction applied at this level) and Plot represents the 12 N-addition subplots nested within MainPlot, with season as repeated measurements within each subplot. F tests were based on the design-based split-plot error strata: whole-plot error (df = 4) for PR, subplot error (df = 4) for NA and NA × PR, and the within-subplot residual (df = 16) for season and its interactions; for this balanced design these tests are identical to the mixed-model F tests when variance components are positive. N_2O flux was natural-log transformed prior to analysis. NA, nitrogen addition (50 kg N ha^{-1} yr^{-1}); PR, precipitation reduction (30% throughfall exclusion). *p* values < 0.001 are reported as "< 0.001"; other p values are rounded to three decimal places.

Table S3. Seasonal variation in soil physicochemical properties and elemental stoichiometric ratios across treatments (mean ± SE).

**Treatment**	**ST (℃)**	**SWC (%)**	**pH**	**TC (mg / g)**	**TN (mg / g)**	**TP (mg / g)**	**SOC (mg / g)**	**TC : TN**	**TC : TP**	**TN : TP**
Spring										
CK	7.25± 0.06cC	44.21± 0.841bA	5.37± 0.01abA	61.50± 0.15abAB	3.98± 0.17aA	0.81± 0.04abA	60.97± 0.98abA	15.22± 0.53aB	74.40± 2.65aB	4.91± 0.14aB
NA	7.22± 0.13cC	47.56± 0.99aA	5.17± 0.05cA	70.10± 3.49aA	4.47± 0.11aB	0.87± 0.03aA	67.91± 1.70aA	15.58± 0.63aA	80.68± 5.22aB	5.17± 0.13aB
PR	7.69± 0.18bC	36.15± 0.97cAB	5.40± 0.01aA	58.66± 2.55bB	3.95± 0.11aB	0.78± 0.05bA	52.36± 1.48cB	14.90± 0.29aA	75.40± 2.75aA	4.95± 0.11aB
NAPR	8.79± 0.04aC	33.30± 0.20cB	5.23± 0.03bcA	59.86± 1.01abAB	3.96± 0.21aA	0.79± 0.01abA	53.48± 1.63bcA	14.90± 0.27aA	76.62± 2.99aB	5.15± 0.26aB
Summer										
CK	16.65± 0.03cA	44.97± 0.57aA	5.30± 0.01abA	56.59± 1.06bB	4.04± 0.48cA	0.82± 0.01abA	49.23± 1.29cB	14.45± 0.64aB	72.57± 2.39aB	4.61± 0.27bB
NA	16.67± 0.03cA	43.27± 0.46aB	5.23± 0.08bcA	77.22± 4.78aA	5.37± 0.30aA	0.91± 0.01aA	68.25± 0.63aA	14.37± 0.11aA	85.25± 5.94aB	5.72± 0.17aB
PR	17.21±	38.44±	5.45±	72.96±	5.01±	0.83±	61.99±	13.04±	89.00±	6.29±

	0.02bA	0.78bA	0.02aA	3.06aA	0.12abA	0.00aA	4.41abA	0.62aB	5.98aA	0.14aA
NAPR	17.80± 0.04aA	35.98± 0.78bA	5.09± 0.04cB	55.11± 0.36bB	4.18± 0.08bcA	0.74± 0.01bAB	57.75± 2.18bA	12.90± 0.17aB	75.63± 3.43aB	5.66± 0.11aAB
Autumn										
CK	14.58± 0.05bB	44.75± 0.71aA	5.14± 0.02aB	65.56± 1.72bA	3.81± 0.21bA	0.60± 0.02bB	57.52± 0.37bA	17.63± 0.10aA	107.52± 0.44abA	6.30± 0.13bcA
NA	14.60± 0.10bB	41.91± 0.90aB	4.95± 0.04bB	77.36± 5.38aA	4.99± 0.29aAB	0.68± 0.01abB	73.75± 2.82aA	15.45± 0.19bA	114.92± 8.02aA	7.33± 0.51aA
PR	13.69± 0.05cB	35.79± 1.00bB	5.25± 0.01aB	65.31± 0.63bAB	3.90± 0.19bB	0.70± 0.02aB	54.74± 2.39bAB	16.22± 0.36abA	89.47± 2.34cA	5.50± 0.07cB
NAPR	15.46± 0.08aB	30.54± 0.61cC	4.94± 0.07bC	63.57± 1.26bA	4.07± 0.21abA	0.67± 0.00abB	55.50± 1.38bA	15.62± 0.58bA	93.10± 3.70bcA	6.33± 0.07bA

Note: Values are means ± SE (n = 3). Different lowercase letters within the same season indicate differences among treatments, whereas different uppercase letters within the same treatment indicate differences among seasons. Grouping letters were derived from Tukey-adjusted comparisons of estimated marginal means from the linear mixed-effects models ($p < 0.05$).

Table S4. Seasonal variation in soil inorganic nitrogen availability and related stoichiometric ratios across treatments (mean ± SE).

Treatment	TIN (mg / kg)	NH_4^+-N (mg / kg)	NO_3^--N (mg / kg)	NO_2^--N (mg / kg)	NH_4^+-N: NO_3^--N	NO_3^--N: NO_2^--N
Spring						
CK	16.77±1.04aB	14.62±0.73bC	1.32±0.06aB	0.25±0.02aA	9.80±0.93bA	5.65±0.55aB
NA	28.42±3.60aC	25.77±0.87aC	4.33±0.16aC	0.23±0.03aA	5.53±0.63cA	20.12±3.47aC
PR	17.40±0.72aB	15.60±0.35abB	1.25±0.21aB	0.21±0.05aA	13.58±1.98aA	4.79±0.75aB
NAPR	25.39±0.28aB	21.73±0.37abC	4.49±0.11aC	0.21±0.02aA	5.18±0.13cA	21.26±2.04aC
Summer						
CK	50.32±2.59bA	45.94±2.56cA	4.52±0.16bB	0.14±0.01aB	11.71±1.49aA	28.30±3.39bB
NA	82.76±7.17aA	69.97±7.13aA	13.27±1.94aB	0.09±0.00aB	5.53±0.81bA	140.98±17.31aB
PR	43.21±1.50bA	35.03±1.55dA	8.06±0.05abA	0.12±0.04aB	4.36±0.21bB	64.92±5.06abAB
NAPR	71.59±3.68aA	58.29±4.51bA	13.19±1.34aB	0.12±0.02aB	5.08±0.35bA	113.40±3.37abB
Autumn						
CK	45.82±2.57bA	34.91±1.28aB	10.81±1.32cA	0.10±0.01aB	3.30±0.30bB	120.81±7.91bA
NA	63.45±6.90aB	43.04±1.31aB	25.31±3.29aA	0.10±0.03aB	2.40±0.66bA	235.50±73.26aA
PR	41.12±1.65bA	36.06±2.14aA	5.00±0.63dAB	0.06±0.02aB	7.62±1.24aB	101.35±7.73bA
NAPR	60.83±2.29aA	42.21±1.38aB	18.54±2.21bA	0.07±0.01aB	2.12±0.17bA	295.03±27.65aA

Note: Values are means ± SE (n = 3). Different lowercase letters within the same season indicate differences among treatments, whereas different uppercase letters within the same treatment indicate differences among seasons. Grouping letters were derived from Tukey-adjusted comparisons of estimated marginal means from the linear mixed-effects models ($p < 0.05$).

Table S5. Seasonal variation in dissolved organic matter, available phosphorus, and DOC-based stoichiometric ratios across treatments (mean ± SE).

Treatment	DOC (mg / kg)	DON (mg / kg)	AP (mg / kg)	DOC: DON	DOC: AP	DON: AP	DOC: NO_3^--N	DOC: NH_4^+-N
Spring								
CK	356.97± 2.44bB	61.55± 4.39aB	13.11± 0.26aC	5.91± 0.38aA	27.43± 0.58bA	4.69± 0.29aB	252.86± 23.01bA	24.08± 1.89abA
NA	483.82± 31.31aB	75.87± 5.89aB	14.90± 0.18aC	6.23± 0.62aA	30.94± 1.83abA	5.27± 0.59aAB	111.95± 9.37cA	20.79± 2.75abA
PR	450.24± 19.51abB	64.28± 3.57aC	11.53± 0.27aC	6.71± 0.88aA	37.01± 3.73aA	5.60± 0.21aA	324.99± 26.83aA	26.47± 1.92aA
NAPR	454.08± 39.20abB	67.52± 6.83aC	13.47± 1.14aC	6.31± 0.34aA	31.66± 1.53abA	5.02± 0.09aB	97.89± 9.02cA	20.43± 2.09bA
Summer								
CK	540.61± 24.75cA	124.44± 5.85bA	37.31± 2.16bA	4.35± 0.11aAB	15.50± 1.31abB	3.55± 0.21aB	139.21± 17.01aB	11.82± 0.11bB
NA	661.69± 22.09bA	180.78± 6.75aA	51.38± 1.71aA	3.72± 0.14aB	12.17± 2.09bB	3.53± 0.78aB	53.72± 13.17bB	9.42± 1.20bB
PR	766.81± 24.24aA	169.15± 12.83aA	42.71± 1.10bA	5.23± 0.59aAB	17.91± 1.22abB	3.50± 0.43aB	91.69± 6.07abB	21.05± 0.74aB
NAPR	672.62± 22.52abA	167.81± 18.72aA	29.94± 2.57cA	4.55± 0.54aB	20.89± 2.29aB	4.74± 0.80aB	50.83± 4.18bAB	11.56± 1.34bB
Autumn								
CK	429.33± 17.55aB	119.23± 2.40bA	19.42± 1.05bB	3.38± 0.10aB	23.57± 1.22aA	7.01± 0.57aA	41.00± 7.12abC	12.24± 1.02aB
NA	463.23± 32.18aB	166.15± 8.20aA	25.65± 2.34aB	3.18± 0.40aB	18.23± 1.23aB	5.84± 0.48aA	26.13± 6.84bB	10.93± 1.11aB
PR	412.21± 8.30aB	110.50± 2.95bB	20.75± 1.67abB	3.73± 0.09aB	20.21± 2.04aB	5.39± 0.42aA	86.65± 11.98aB	11.49± 0.47aC
NAPR	422.63± 32.74aB	137.29± 2.24abB	21.56± 1.34abB	3.08± 0.20aB	21.66± 3.39aB	6.98± 0.82aA	24.74± 2.06bB	10.04± 0.90aB

Note: Values are means ± SE (*n* = 3). Different lowercase letters within the same season indicate differences among treatments, whereas different uppercase letters within the same treatment indicate differences among seasons. Grouping letters were derived from Tukey-adjusted comparisons of estimated marginal means from the linear mixed-effects models ($p < 0.05$).

Table S6. Seasonal variation in N-transformation-related enzyme indicators across treatments (mean ± SE).

Treatment	UE μg /d /g	AMO (U / l)	NXR (U / l)	NR μmol /d /g	NIR μmol /d /g	NOR (U / l)	N_2OR (U / l)
Spring							
CK	304.28± 12.08abA	20.62± 0.34aC	57.92± 0.37aC	0.63± 0.00bcA	39.62± 0.35bA	260.29± 0.13aC	141.96± 0.54aC
NA	320.34± 11.16aB	17.93± 0.51bC	40.23± 0.29cC	0.65± 0.00abB	41.31± 0.08bA	200.94± 0.40cC	117.11± 0.44cC
PR	228.11± 3.04cC	15.47± 0.20cC	30.22± 0.33dC	0.62± 0.00cA	39.61± 0.64bA	175.22± 0.13dC	104.34± 0.51dC

NAPR	284.75± 1.91bB	18.79± 0.03bC	49.05± 0.43bC	0.65± 0.00aA	43.61± 0.51aA	228.22± 0.10bC	130.74± 0.28bC
Summer							
CK	297.87± 3.81cA	46.16± 0.42aA	64.31± 0.38aA	0.58± 0.01cB	34.43± 0.04cB	273.38± 0.16aA	148.50± 0.15aA
NA	382.30± 10.34aA	42.82± 0.33cA	45.87± 0.38cA	0.65± 0.01aAB	37.84± 0.41abB	221.45± 0.27cA	125.77± 0.55cA
PR	303.99± 2.27cA	40.60± 0.50dA	36.87± 0.38dA	0.61± 0.01bAB	36.02± 0.56bcB	191.23± 0.15dA	112.36± 0.49dA
NAPR	334.17± 0.76bA	44.52± 0.16bA	55.08± 0.28bA	0.64± 0.01aA	39.89± 0.72aB	249.66± 0.07bA	138.39± 0.50bA
Autumn							
CK	292.93± 5.02bA	32.55± 0.33aB	59.93± 0.89aB	0.62± 0.00bA	30.20± 0.83cC	267.81± 0.11aB	144.74± 0.18aB
NA	325.47± 6.75aB	28.70± 0.60cB	43.13± 0.40cB	0.67± 0.01aA	32.87± 0.27bC	214.08± 0.28cB	122.01± 0.25cB
PR	261.99± 0.94cB	26.81± 0.23dB	33.39± 0.34dB	0.59± 0.00cB	30.50± 0.98cC	185.58± 0.11dB	108.95± 0.36dB
NAPR	315.54± 2.26abA	30.45± 0.55bB	52.24± 0.25bB	0.61± 0.00bB	35.75± 0.36aC	242.59± 0.25bB	135.07± 0.24bB

Note: Values are means ± SE (n = 3). Different lowercase letters within the same season indicate differences among treatments, whereas different uppercase letters within the same treatment indicate differences among seasons. Grouping letters were derived from Tukey-adjusted comparisons of estimated marginal means from the linear mixed-effects models ($p < 0.05$).

Table S7. Summary statistics and diagnostics for the redundancy analysis.

Part A. Overall RDA model.

N	R^2	Adjusted R^2	F	*p*	RDA1 percent	RDA2 percent
36	0.5969	0.4961	5.92	0.001	52.72	26.81

Part B. Predictor diagnostics.

Indicator	VIF	Envfit R^2
SWC	1.39	0.09
ST	7.33	0.56
pH	2.36	0.14
SOC	1.68	0.58
AP	4.73	0.50
DOC : DON	2.59	0.37
DOC : NO_3^-	3.32	0.41

Table S8. Season-adjusted associations between N-transformation-related enzyme indicators and soil N_2O flux.

Indicator	Standardized β	95% CI	*p*
AMO	-1.053	(-3.158, 1.052)	0.307
NXR	-0.094	(-0.604, 0.416)	0.692
NR	-0.003	(-0.226, 0.220)	0.978
NIR	-0.557	(-1.129, 0.015)	0.056
NOR	-0.176	(-0.691, 0.340)	0.467
N_2OR	-0.106	(-0.619, 0.406)	0.654
UE	-0.008	(-0.305, 0.289)	0.958

Note: Separate linear mixed-effects models were fitted for each N-transformation-related enzyme indicator. In each model, z-score-standardized ln-transformed soil N_2O flux was used as the response, the corresponding standardized enzyme indicator as the focal predictor, season as a categorical fixed effect, and plot as a random intercept. β denotes the standardized regression coefficient, and values in parentheses indicate 95% confidence intervals. The analysis included 36 observations from 12 plots. *p* values were obtained using Satterthwaite-approximated degrees of freedom.

Table S9. Predictive performance of the random-forest model under out-of-bag and grouped cross-validation.

Validation	N	R^2	RMSE	MAE	Role
OOB diagnostic	36	0.829	0.124		Model-internal diagnostic
Leave-one-subplot-out grouped CV	36	0.837	0.121	0.088	Repeated-measure sensitivity
Leave-one-main-plot-out grouped CV	36	0.828	0.124	0.091	Primary conservative validation

Note: The random-forest model included nine predictors (SWC, pH, SOC, DOC:DON, DOC:NO_3^-, UE, AMO, NIR, and N_2OR) and was fitted with 2000 trees, mtry = 3, and nodesize = 5. Out-of-bag performance is reported as a model-internal diagnostic. Leave-one-subplot-out cross-validation retained all seasonal observations from the same subplot within the same fold, whereas leave-one-main-plot-out cross-validation additionally withheld the paired N-addition and control subplots within each precipitation main plot and was used as the primary conservative validation. For the grouped cross-validation procedures, R^2, RMSE, and MAE were calculated from pooled out-of-fold predictions.

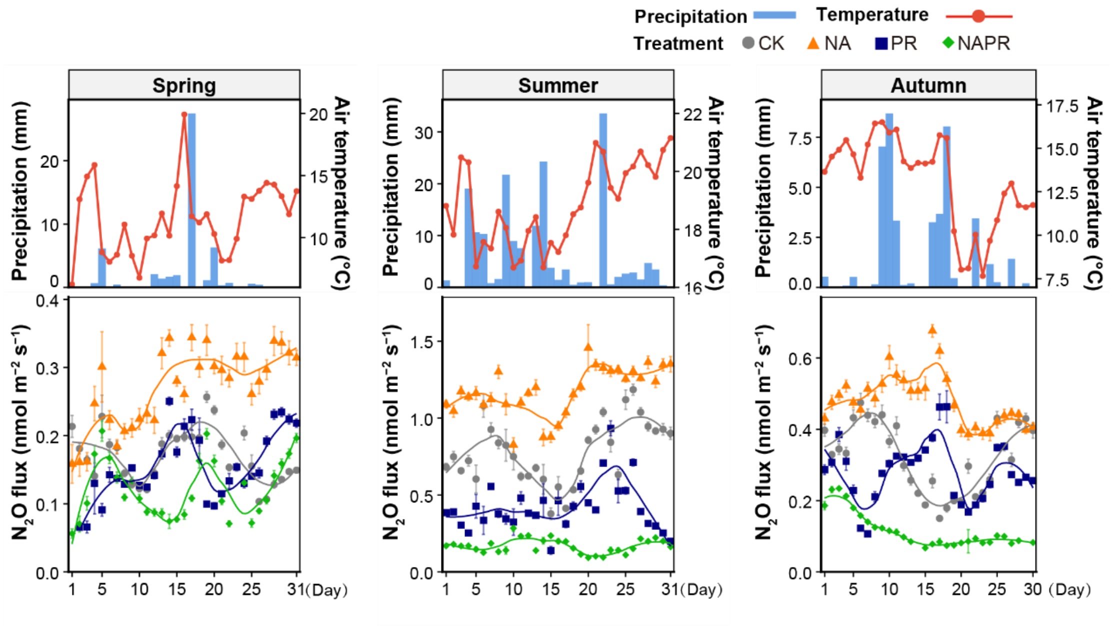

Figure S1. Daily environmental conditions and soil N_2O flux dynamics during the three measurement periods.

Note: The upper panels show daily total precipitation and air temperature during the spring, summer, and autumn measurement periods. Daily precipitation was derived from the ERA5-Land reanalysis dataset, and air temperature was calculated from chamber-temperature records. The lower panels show daily soil N_2O fluxes for CK, NA, PR, and NAPR treatments. Points represent daily means, error bars represent standard errors calculated across hourly mean observations within each day, and smooth lines illustrate temporal trends. The N_2O error bars therefore characterize within-day temporal variability rather than biological replicate variation among the three experimental plots. CK, control; NA, nitrogen addition; PR, precipitation reduction; NAPR, combined nitrogen addition and precipitation reduction.

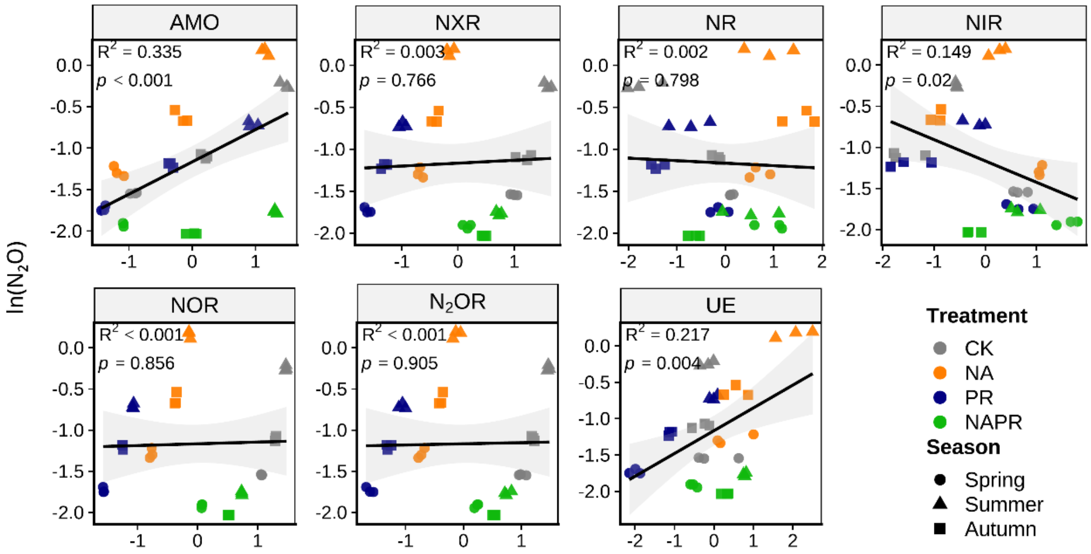

Figure S2. Pooled relationships between soil N-transformation-related enzyme indicators and ln-transformed N_2O flux**.**

Note: Each panel shows the pooled linear relationship between ln-transformed soil N_2O flux and one enzyme indicator across all samples. Points represent subplot-level observations, with colors indicating treatments and symbols indicating seasons. Solid lines denote ordinary least-squares regression fits, and shaded bands indicate 95% confidence intervals. The R^2 and *p* values shown in each panel are from simple linear regressions.
